# Elevated LILRB1 expression predicts poor prognosis and is associated with tumor immune infiltration in patients with glioma

**DOI:** 10.1186/s12885-023-10906-2

**Published:** 2023-05-04

**Authors:** Renheng Zou, Xunlong Zhong, Kairong Liang, Cheng Zhi, Danmin Chen, Zhichao Xu, Jingbai Zhang, Degui Liao, Miaoling Lai, Yuhao Weng, Huaidong Peng, Xiao Pang, Yunxiang Ji, Yanbin Ke, Hongri Zhang, Zhaotao Wang, Yezhong Wang

**Affiliations:** 1 Institute of Neuroscience, Department of Neurosurgery, the Second Affiliated Hospital of Guangzhou Medical University, Guangzhou, 510260 China; 2 Science and Technology Innovation Center, Institute of Clinical Pharmacology, Guangzhou University of Chinese Medicine, Guangzhou, 510405 China; 3 Department of Pharmacy, the Second Affiliated Hospital of Guangzhou Medical University, Guangzhou, 510260 China; 4 Department of Neurosurgery, The First Affiliated Hospital, College of Clinical Medicine, Henan University of Science and Technology, Luoyang, 471003 Henan China

**Keywords:** LILRB1, Immune infiltration, M2 macrophage, Methylation, Immune checkpoint, Glioma

## Abstract

**Background:**

Leukocyte immunoglobulin-like receptor subfamily B1 (LILRB1) is regarded as an inhibitory molecule. However, the importance of LILRB1 expression in glioma has not yet been determined. This investigation examined the immunological signature, clinicopathological importance and prognostic value of LILRB1 expression in glioma.

**Methods:**

We used data from the UCSC XENA database, the Cancer Genome Atlas (TCGA) database, the Chinese Glioma Genome Atlas (CGGA) database, the STRING database, the MEXPRESS database and our clinical glioma samples to perform bioinformatic analysis and used vitro experiments to examine the predictive value and potential biological roles of LILRB1 in glioma.

**Results:**

Higher LILRB1 expression was considerably present in the higher WHO grade glioma group and was linked to a poorer prognosis in patients with glioma. Gene set enrichment analysis (GSEA) revealed that LILRB1 was positively correlated with the JAK/STAT signaling pathway. LILRB1 combined with tumor mutational burden (TMB) and microsatellite instability (MSI) may be a promising indicator for the effectiveness of immunotherapy in patients with glioma. Increased LILRB1 expression was positively linked with the hypomethylation, M2 macrophage infiltration, immune checkpoints (ICPs) and M2 macrophage makers. Univariate and multivariate Cox regression analyses determined that increased LILRB1 expression was a standalone causal factor for glioma. Vitro experiments determined that LILRB1 positively enhanced the proliferation, migration and invasion in glioma cells. MRI images demonstrated that higher LILRB1 expression was related with larger tumor volume in patients with glioma.

**Conclusion:**

Dysregulation of LILRB1 in glioma is correlated with immune infiltration and is a standalone causal factor for glioma.

**Supplementary Information:**

The online version contains supplementary material available at 10.1186/s12885-023-10906-2.

## Introduction

Gliomas are the most frequent primary intracranial tumors and account for 81% of malignant intra-cerebral tumors including low grade gliomas (LGG) (World Health Organization (WHO) grade II and grade III) and high grade gliomas (HGG) (glioblastomas (GBM)) [1, 2]. Despite aggressive surgery, chemotherapies with temozolomide, and radiation, the average survival remains short. Therefore, there is an urgent need for a new and efficient therapeutic approach to treat gliomas.

A transmembrane glycoprotein leukocyte immunoglobulin-like receptor subfamily B1 (LILRB1), also called CD85j, LIR1 and ILT2 [3], serves as a crucial receptor for the human leukocyte antigen G (HLA-G)[4]. Four immunoreceptor tyrosine-type inhibition motifs found in LILRB1’s intracellular domain have the ability to decrease cell activity and deliver inhibitory signals to cells [5]. It is regarded as an immunosuppressive receptor and expressed on several different types of human immune cell types, including dendritic cells, monocytes, T cells, B cells, subsets of NK cells and macrophages [6, 7]. A signal that lowers the immune response is transmitted by human major histocompatibility complex (MHC) class I molecules, which are LILRB1 ligands [8]. LILRB1 inhibits the immune system by combining conventional and unconventional MHC components. Specifically, it regulates prenatal immunological tolerance and induces immune tolerance to transplants [8]. The immune system’s ability to sneak up on tumor cells is a major factor in their rapid growth [9]. LILRB1 has been demonstrated to be crucial in promoting tumor development and metastasis. For instance, Fan J et al. showed that compared with healthy donors, patients with hepatocellular carcinoma had higher LILRB1 expression in granulocytes from peripheral blood [10]. Y. Zhang et al. determined that LILRB1 was overexpressed and was closely associated to the differentiation degree of gastric cancer [11]. However, little is understood about LILRB1’s probable biological role in glioma.

In order to achieve this, we used bioinformatics and vitro experiments to examine the predictive importance and potential biological roles of LILRB1 in glioma.

## Method and materials

### Expression analysis

We validated LILRB1 mRNA expression levels for various tumors using data from the UCSC XENA database (https://xenabrowser.net/datapages/) ( TCGA and GTEx ), and we evaluated LILRB1 mRNA expression levels for various glioma subtypes using data from the Cancer Genome Atlas (TCGA) database ( https://portal.gdc.cancer.gov ) (TCGA-GBM and TCGA-LGG) and the Chinese Glioma Genome Atlas (CGGA) database (http://www.cgga.org.cn/analyse/RNA-data.jsp). We investigated the association between LILRB1 expression and the clinicopathological characteristics of our clinical glioma using the Fisher test and T test. We looked into the link between LILRB1 expression and the WHO grades of our clinical glioma using Kruskal-Wallis test. We analyzed and visualized the data from UCSC XENA, TCGA, the CGGA and our clinical glioma using R software (v 3.6.3).

### Survival analysis

Using the R survival and survminer tools, we incorporated the data from TCGA (https://portal.gdc.cancer.gov) (TCGA-GBM and TCGA-LGG), the CGGA (http://www.cgga.org.cn/analyse/RNA-data.jsp) and our clinical glioma to do a Kaplan–Meier survival analysis. From the TCGA dataset and the CGGA, where the technique of collection and utilization agreed with the policies and guidelines, raw counts of RNA-sequencing data (level 3) and related clinical data from LILRB1 were collected. The Kaplan-Meier survival analysis and log-rank test were also used for comparing the survival rates of the two groups. Hazard ratios (HR) with 95% confidence intervals (CI) and P -values were computed using univariate Cox proportional hazards regression, log-rank testing and Kaplan-Meier curves. We used R software 3.6.3 and R packages to carry out all of the aforementioned analysis techniques.

### Co-expressed genes of LILRB1 and gene set enrichment analysis

We used the TCGA dataset (https://portal.gdc.cancer.gov) (TCGA-GBM and TCGA-LGG) for obtaining co-expression genes of LILRB1 and for carrying out gene set enrichment analysis (GSEA)[12] using the Clusterprofiler package [13]. A heat map was used to show the top 50 genes that were either favorably or negatively correlated. Using the ggplot2 R package (v 3.3.3), a volcano plot performed by enrichment was considered significant if |log2(FC)| > 1 and P-value < 0.05. We conducted enrichment analysis of hub genes using Kyoto Encyclopedia of Genes and Genomes (KEGG) pathways (https://www.kegg.jp/kegg/kegg1.html) and Gene Ontology (GO) keywords (molecular function (MF), cellular component (CC) and biological process (BP) categories). Meanwhile, we used the Clusterprofiler program to perform GSEA to look for biological pathways that were markedly distinct between the LILRB1 high and LILRB1 low groups. In MSigDB Collections (https://www.gsea-msigdb.org/gsea/msigdb/index.jsp) (C2.CP), we ran studies with a number of size 3 and 10,000 simulations. If the false discovery rate (FDR) < 0.25 and the p.adjust < 0.05, enrichment was judged significant.

### Protein-protein interaction network analysis

We located the genes and proteins that interact with LILRB1 both physically and functionally by using the STRING database (https://string-db.org). Additionally, the top 10 hub genes of LILRB1 identified by the CytoHubba plugin were ranked according to the normalized cross correlation (NCC) score, and a combined score > 0.9 (high confidence) was used to build the protein-protein interaction (PPI) network, which was then further displayed using Cytoscape.

### DNA methylation analysis

Using the MEXPRESS database (https://mexpress.be) for TCGA-LGG and TCGA-GBM, we examined LILRB1 methylation. We investigated the connection between DNA methylation and LILRB1 expression using Pearson correlation analysis. For various methylation locations, correlation coefficients (R) and Benjamini-Hochberg-adjusted P-values were found. Using the MethSurv program (https://biit.cs.ut.ee/methsurv), we were able to visualize LILRB1 methylation and the Kaplan-Meier-based connection between LILRB1 hyper/hypomethylation and overall survival (OS).

### Tumor mutational burden and microsatellite instability analysis

From the TCGA dataset (https://portal.gdc.cancer.gov) (TCGA-GBM and TCGA-LGG), we downloaded RNA-sequencing expression (level 3) profiles and related clinical data for glioma. A correlation analysis between LILRB1 expression and TMB/MSI was carried out using Spearman’s method. We used Spearman’s correlation analysis to explain the relationship between quantitative variables without a standard deviation.

### Tumor infiltration analysis

We used the R GSVA package [14] based on TCGA (https://portal.gdc.cancer.gov) (TCGA-GBM and TCGA-LGG) to perform the single-sample GSEA (ssGSEA) to estimate the tumor infiltration of 24 immune cell types. We were able to gather feature gene panels for every type of immune cell from an earlier publication [15]. After that, we evaluated the relationship between LILRB1 expression and the infiltration of B cell, CD4^+^T cell, macrophage, neutrophil and dendritic cell neutrophils using the TIMER database (https://cistrome.shinyapps.io/timer/). The cutoff for a meaningful relationship between LILRB1 and immune cell infiltration was a P-value < 0.01. Using the TIMER2.0 databases (http://timer.cistrome.org), OS was analyzed as a function of LILRB1 expression in B cell, CD4^+^T cell, macrophage M2, neutrophil and myeloid dendritic cell.

### Univariate and multivariate Cox regression analysis

With TCGA data (https://portal.gdc.cancer.gov) (TCGA-GBM and TCGA-LGG), we used univariate and multivariate Cox analysis to investigate the relationship between LILRB1 expression and other clinicopathological variables (age, gender, race, WHO grade, and isocitrate dehydrogenase (IDH) status) on OS, progression-free interval (PFI) and disease-specific survival (DSS). The cut-off point was chosen at a P-value < 0.05. The P-value, 95% CI and HR of each variable were determined using the R forestplot tool.

### Patients and sample

We collected samples of tumor and surrounding tissues from 38 patients in the Second Affiliated Hospital of Guangzhou Medical University, who had undergone curative surgery from 2020 to 2022 in our hospital. This work was accepted by the Institutional Ethics Committee in the Second Affiliated Hospital of Guangzhou Medical University (2020-YJS-KS-01). The tumor tissues of the 38 patients were used for immunohistochemistry to examine the expression of LILRB1.Proteins (24 pairs) were isolated from frozen tumor tissues and adjacent tissues for western blotting assay.

### Western blot

We performed Western blot using clinically normal and tumor tissues homogenates. Using PRO-PREP™ Protein Extraction Solution (Cell/Tissue) (iNtRON Biotechnology, Korea) and Pierce™ BCA Protein Assay Kit (Thermo Fisher Scientific Inc.), proteins were extracted and measured directed by the manufacturer’s guidelines. On 10% sodium dodecyl sulfate-polyacrylamide gel electrophoresis (SDS-PAGE), appropriate amounts of protein (30 µg) were separated and then transferred to polyvinylidene difluoride membranes (Merck, KGaA, Darmstadt, Germany). The membranes were treated with rabbit anti-LILRB1 (1:1000, ab238145, Abcam), mouse Anti-GAPDH (1:2000, TA-08, ZSGB-BIO) overnight at 4 ℃ after being blocked with 5% bovine serum albumin at ambient temperature for 1 h. Immobilon Western HRP Substrate (Merck, KGaA, Darmstadt, Germany) was used to detect the signal after an hour of incubation at room temperature with the relevant secondary antibodies. The expression of LILRB1 were measured by ImageJ.

### Cell lines

U87 and U251 cells were bought from iCell Bioscience Inc (Shanghai, China). Human microglia clone 3 (HMC3) cell was purchased from Procell Life Science & Technology Co., Ltd. We cultured U87, U251 and HMC3 cells in Dulbecco’s Modified Eagle Medium (DMEM) (Thermo Fisher Scientific, Inc.) + 10% fetal bovine serum (FBS) (Thermo Fisher Scientific, Inc.) at 37 ℃, 5% CO_2_ incubator.

### Lentivirus transfection

We acquired lentiviral shRNA constructs from GeneChem Co., Ltd., Shanghai, China. As directed by the manufacturer, the lentiviral particles were transfected into HMC3 cells. Cells for further investigation were collected 48 h after the transfection.

### CCK-8 assay

The constructed Control, sh LILRB1#1, sh LILRB1#2 HMC3 cells were sown in the upper chamber and U87, U251cells were sown in the lower chamber. They were respectively co-cultured in 24 well culture plates with 0.4 μm Pore Polycarbonate Membrane (Corning Inc.) (2 × 10^4^/well) at 37 ℃, 5% CO_2_ incubator. The old solution was discarded at 24 h, 48 and 72 h. The lower chamber cells were then washed twice with Phosphate Buffered Saline (PBS) (Servicebio), 700 µl DMEM + 10% Cell Counting Kit-8 (CCK-8) solution (Beyotime) was added, and the mixture was cultivated at 37 ℃, 5% CO_2_ incubator for 2 h. Subsequently, 100 µl culture medium was added to each well of the 96-well plate to be tested. We measured the optical density (OD) of each experimental well at 450 nm using a multimode reader, and we looked for variations in each group’s capacity for cell proliferation.

### Transwell assay

The constructed control, sh LILRB1#1, sh LILRB1#2 HMC3 cells were sown in the lower chamber containing 600 µl of complete medium (DMEM and 20% FBS) and U87, U251 cells were put in the upper chamber with Matrigel (Corning Inc.) or without Matrigel containing serum-free DMEM. They were respectively co-cultured in 24 well culture plates with 8 μm Pore Polycarbonate Membrane (Corning Inc.) (2 × 10^4^/well) at 37 ℃, 5% CO_2_ incubator. The cells in the upper chamber were co-cultured for 48 h before being fixed for 15 min with 4% glutaraldehyde (Sangon Biotech (Shanghai) Co.,Ltd.) and stained for 5 min with 1% crystal violet (Beyotime) at ambient temperature. The cells that had not migrated through the well were taken out using a cotton swab. Cells were photographed (magnification, ×100) and counted.

### Immunohistochemistry

Deparaffinized tissue slices were heated in a microwave, immersed in 0.01 M sodium citrate buffer (pH 6.0), and then incubated with antibodies against LILRB1 (1:2000; ab170909, Abcam) for 12 h at 4 °C. The following day, tissue sections were mounted after being dried, counterstained with hematoxylin, and reacting with secondary antibodies and 3, 3’-diaminobenzidine. Tissue sections were photographed (magnification, ×100). The immunohistochemistry’s integrated optical density (IOD) value of LILRB1 were measured by Image-Pro Plus.

### MRI image analysis

We obtained the MRI images of 38 patients from the Second Affiliated Hospital of Guangzhou Medical University. The Wilcoxon rank sum test was used to examine the relationship between LILRB1 expression and tumor volume. Using the T test, we investigated the relationship between LILRB1 expression and tumor spread distance.

### Statistical analysis

All of the data were examined using the SPSS 25 program and are composites of three independent studies. Simple comparisons between two groups were made using T test or the Wilcoxon rank sum test, and multiple comparisons between the groups were assessed using either the One-way ANOVA test or the Two-way ANOVA test. R software 3.6.3 was used to evaluate and visualize the analytical process. A P-value < 0.05 was regarded as statistically significant.

## Results

### LILRB1 was overexpressed in glioma and associated with poor prognosis in patients with glioma

**Fig. 1:**
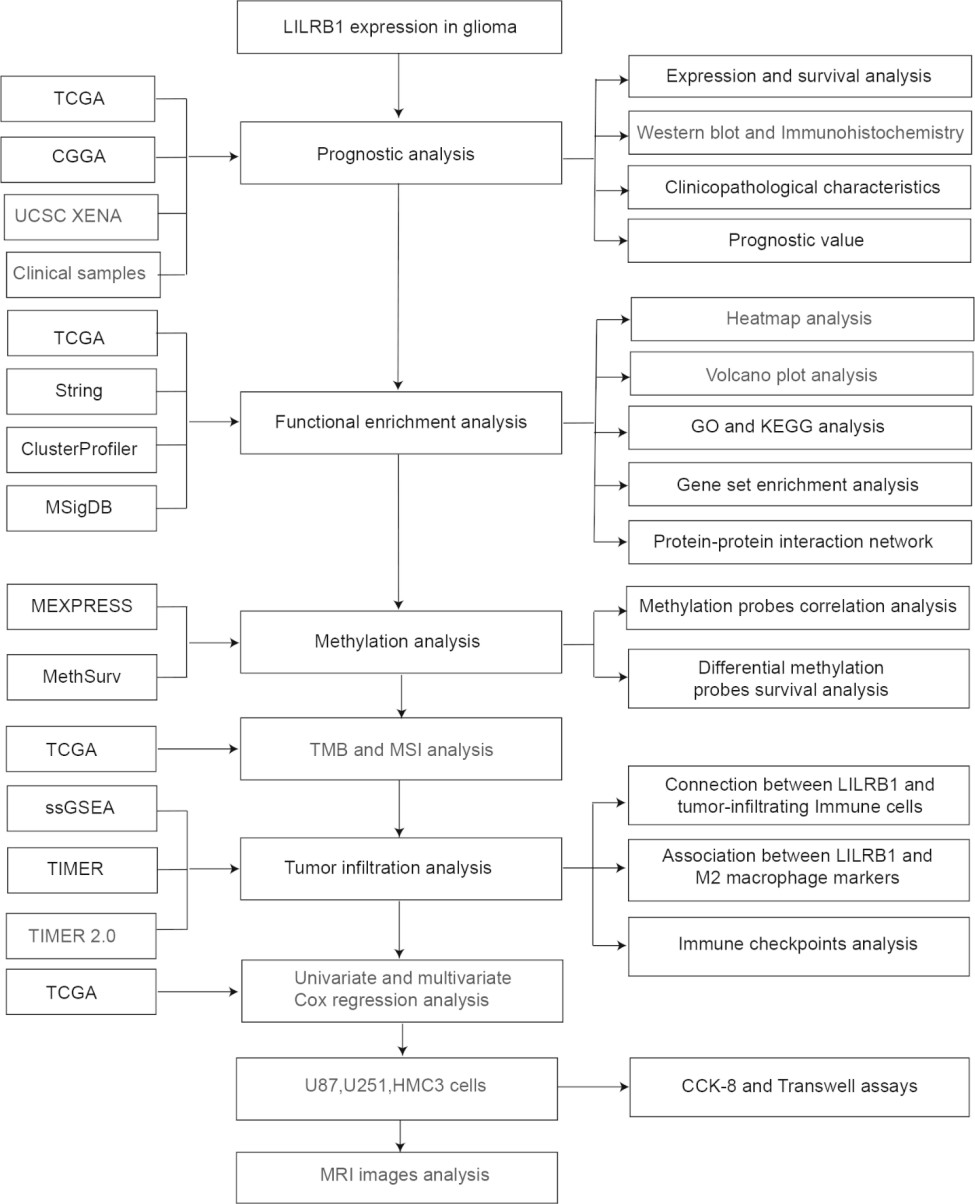
Flowchart of the study

**Fig. 2:**
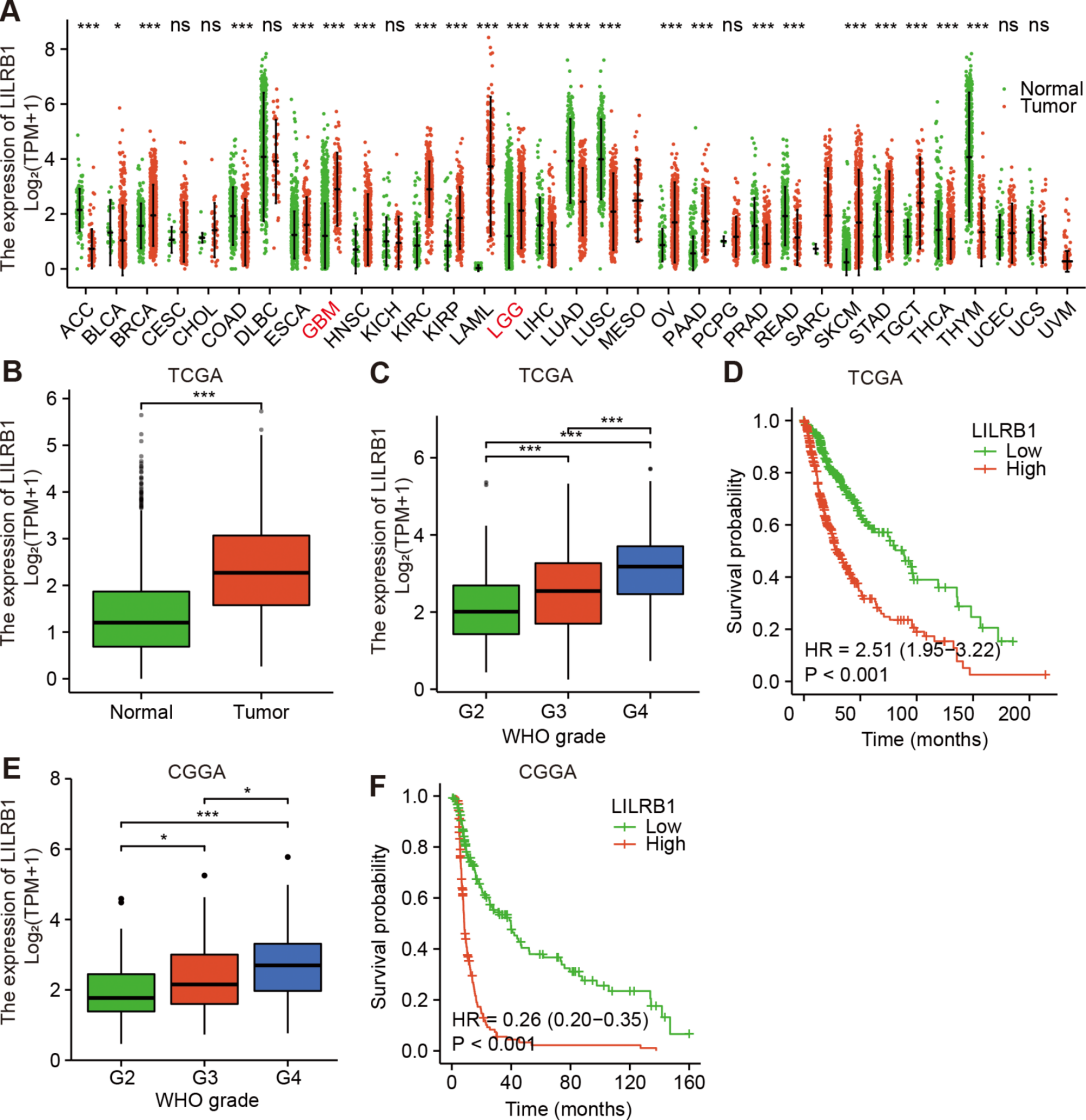
Differential LILRB1 expression levels in all tumors and correlation with survival in glioma. Differential LILRB1 expression in all cancers between the tumor and adjacent normal tissues (**A**) and patients with glioma (**B**) in TCGA. The association between tumor grade and LILRB1 in patients with glioma (**C, E**). Kaplan-Meier curves of patients with glioma sorted by LILRB1 expression (**D, F**). ns, P ≥ 0.05; *, P < 0.05; ***, P < 0.001

**Fig. 3:**
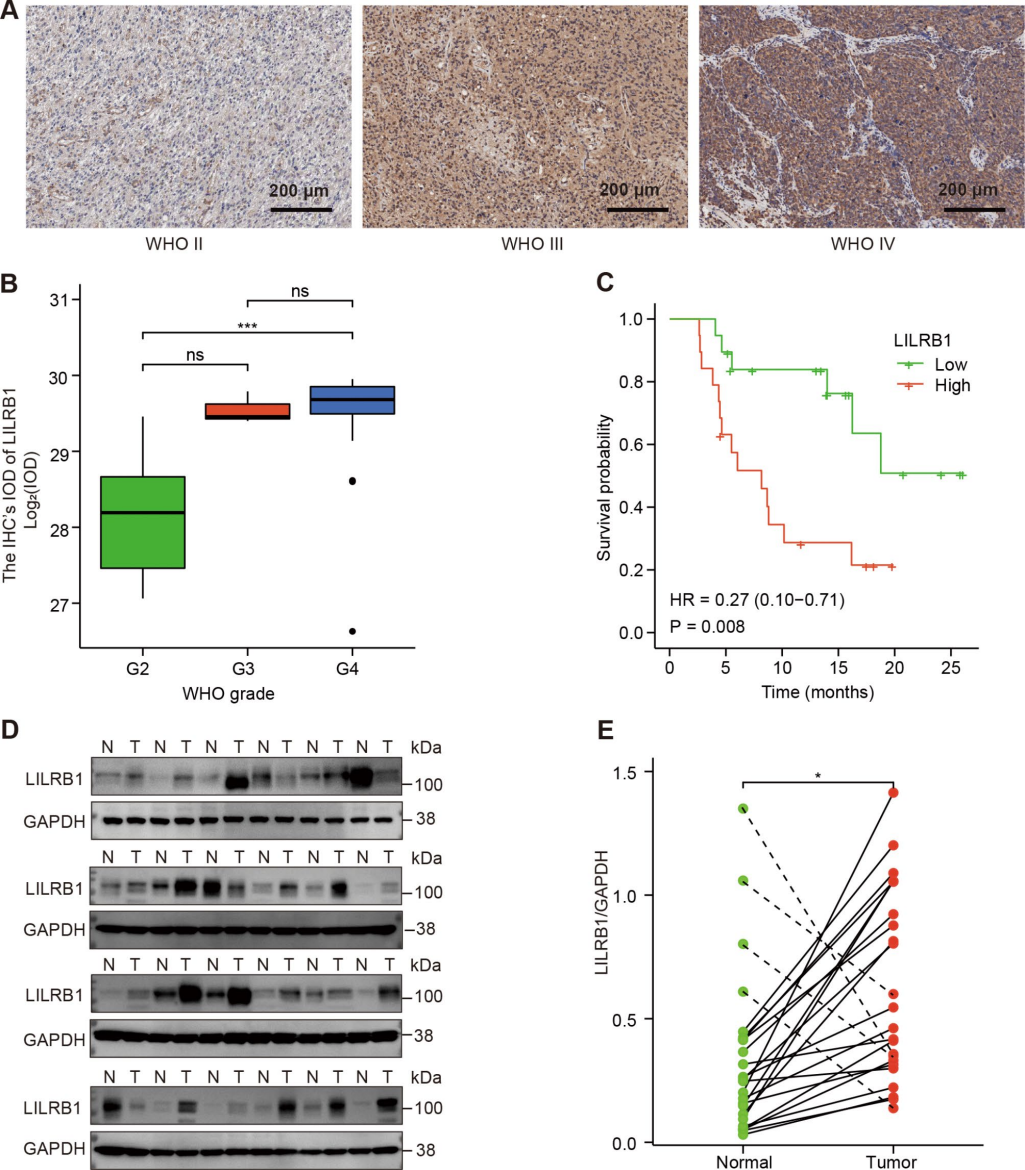
The LILRB1 expression in different grade of clinical glioma samples. The LILRB1 expression in different grade of glioma were statistically analyzed in 38 glioma tissues (**A-B**). Survival curves for overall survival sorted by LILRB1 expression in glioma tissues derived from 38 patients with glioma (**C**). The western blot results of LILRB1 expression in 24 patients with glioma (**D-E**). ns, P ≥ 0.05; *, P < 0.05; ***, P < 0.001

Fig. 1 depicted the study’s flowchart. To explore the possible role of LILRB1, we examined the expression of the LILRB1 gene in different cancers by using data from UCSC XENA. Compared with normal samples, LILRB1 was significantly overexpressed in GBM and LGG (Fig. 2A). In comparison to normal samples, the expression of the LILRB1 gene in glioma was significantly overexpressed (Fig. 2B). We examined the data from the CGGA and TCGA databases to ascertain the link between LILRB1 and clinicopathological traits in patients with glioma. Increased LILRB1 expression was linked with more advanced tumor grades (P < 0.001) (Fig. 2C, E). Using TCGA and the CGGA database, it was determined how LILRB1 expression affected the survival of patients with glioma. Patients with gliomas that expressed a lot of LILRB1 had significantly lower survival times. (P < 0.001) (Fig. 2D, F). In accordance with the results in the TCGA portal and CGGA database, LILRB1 was upregulated in glioma tissues and positively associated with tumor progression and poor prognosis in our 38 clinical samples. Immunohistochemical staining of the 38 clinical samples confirmed a different level of LILRB1 expression in tumor tissues in different glioma grades (Fig. 3A) and the LILRB1 expression increased with rising glioma pathological grade (Fig. 3B). In addition, we performed survival curves for OS stratified by LILRB1 expression in glioma tissues derived from the 38 glioma samples (Fig. 3C). Additionally, LILRB1 was overexpressed in glioma tissues in our sample of 24 paired tumor and peritumor tissues from patients with glioma (Fig. 3D-E).

### Clinical correlation of LILRB1 in glioma

Elevated LILRB1 in glioma was substantially correlated with the increasing WHO grade (G4 vs. G2&G3, OR = 4.172, 95%CI [2.827–6.263], P < 0.001), IDH status (WT vs. Mut, OR = 4.275, 95%CI [3.060–6.026], P < 0.001), 1p/19q codeletion (non-codel vs. codel, OR = 12.506, 95%CI [7.762–21.164], P < 0.001), Age (> 60 vs.<= 60, OR = 1.738 ,95%CI [1.198–2.539], P = 0.004) (Table 1). While neither a significant relationship with race nor gender was seen in the TCGA (Table 2), elevated LILRB1 in our clinical glioma was highly connected with WHO grade and IDH (Table 3). Further study using logistic regression analysis revealed a connection between increased LILRB1 expression and worse prognosis in patients with glioma. According to these findings, LILRB1 acts as an oncogene in glioma, and high expression of this gene predicts a poor prognosis.

**Table 1:** LILRB1 expression was linked with clinicopathological characteristics in TCGA.

Characteristics	Total(N)	Odds Ratio (OR)	P value
WHO grade (G4 vs. G2&G3)	635	4.172 (2.827–6.263)	< 0.001
1p/19q codeletion (non-codel vs. codel)	689	12.506 (7.762–21.164)	< 0.001
IDH status (WT vs. Mut)	686	4.275 (3.060–6.026)	< 0.001
Gender (Male vs. Female)	696	1.358 (1.005–1.836)	0.047
Age (> 60 vs. <=60)	696	1.738 (1.198–2.539)	0.004

**Table 2:** The expression profile of LILRB1 mRNA and clinicopathological glioma risk variables in TCGA.

Characteristic	Low expression of LILRB1	High expression of LILRB1	p
n	348	348	
WHO grade, n (%)			< 0.001
G2	149 (23.5%)	75 (11.8%)	
G3	119 (18.7%)	124 (19.5%)	
G4	41 (6.5%)	127 (20%)	
IDH status, n (%)			< 0.001
WT	69 (10.1%)	177 (25.8%)	
Mut	275 (40.1%)	165 (24.1%)	
1p/19q codeletion, n (%)			< 0.001
codel	151 (21.9%)	20 (2.9%)	
non-codel	195 (28.3%)	323 (46.9%)	
Gender, n (%)			0.055
Female	162 (23.3%)	136 (19.5%)	
Male	186 (26.7%)	212 (30.5%)	
Race, n (%)			0.959
Asian	6 (0.9%)	7 (1%)	
Black or African American	16 (2.3%)	17 (2.5%)	
White	317 (46.4%)	320 (46.9%)	
Age, n (%)			0.005
<=60	292 (42%)	261 (37.5%)	
> 60	56 (8%)	87 (12.5%)	

**Table 3:** The expression profile of LILRB1 mRNA and clinicopathological glioma risk variables in our clinical data

Characteristics	Low expression of LILRB1	High expression of LILRB1	P value
n	19	19	
WHO grade, n (%)			< 0.001
G2	11 (28.9%)	0 (0%)	
G3	2 (5.3%)	1 (2.6%)	
G4	6 (15.8%)	18 (47.4%)	
IDH status, n (%)			0.007
Mut	12 (31.6%)	3 (7.9%)	
WT	7 (18.4%)	16 (42.1%)	
Gender, n (%)			1.000
Female	9 (23.7%)	9 (23.7%)	
Male	10 (26.3%)	10 (26.3%)	
Age, n (%)			0.079
> 60	3 (7.9%)	9 (23.7%)	
<=60	16 (42.1%)	10 (26.3%)	

### Co-expressed genes of LILRB1 and gene set enrichment analysis in glioma

**Fig. 4:**
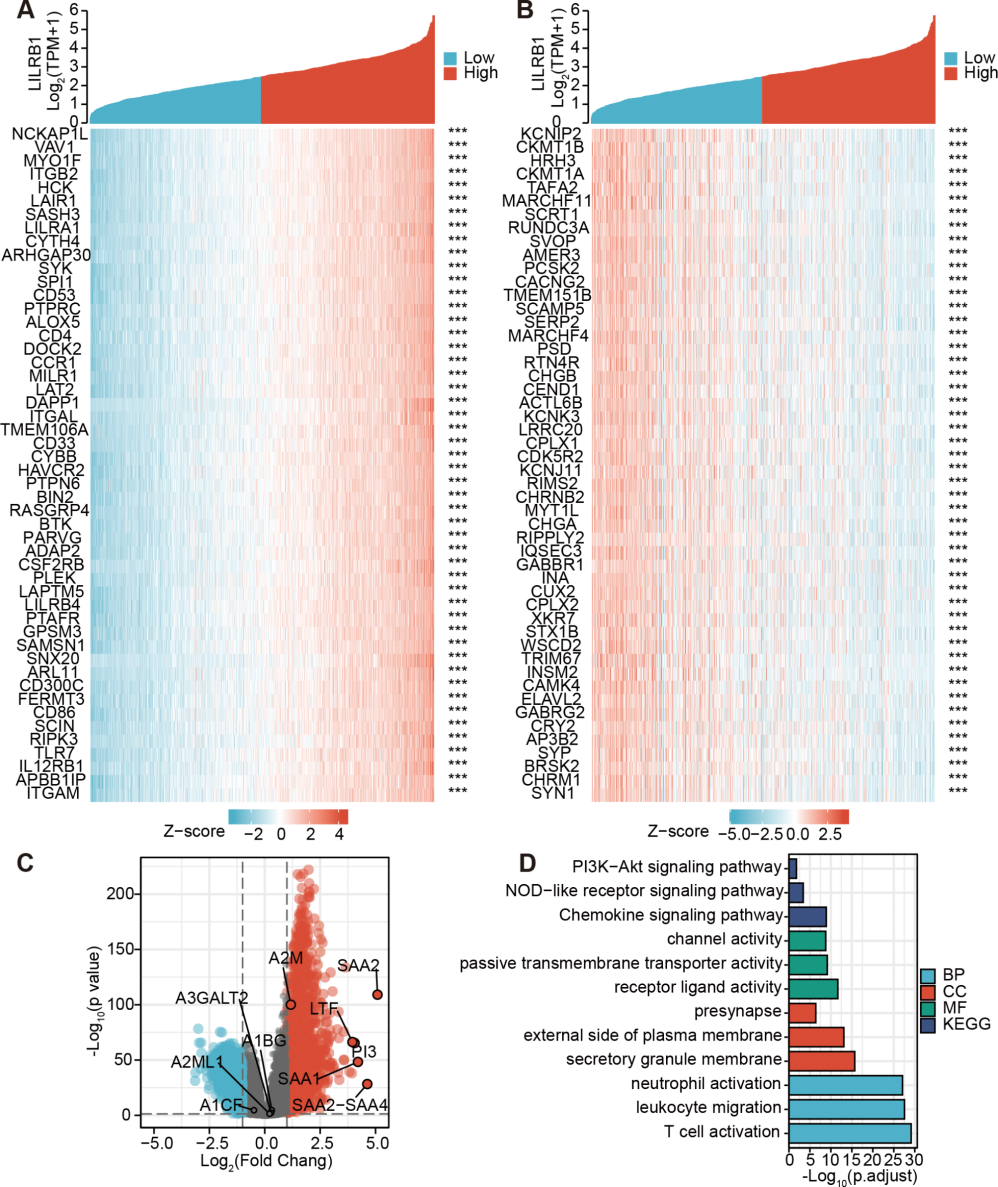
Co-expression analysis and functional enrichment analysis in glioma. The heatmap of the top 50 genes of LRLRB1 that are connected positively and negatively in glioma (**A-B**). The Pearson positive and negative association finding of LRLRB1 in glioma is shown in a volcano plot (**C**). GO keywords and KEGG pathways of the overlapping differentially expressed genes in glioma are represented in a bar graph (**D**). ***, P < 0.001

**Fig. 5:**
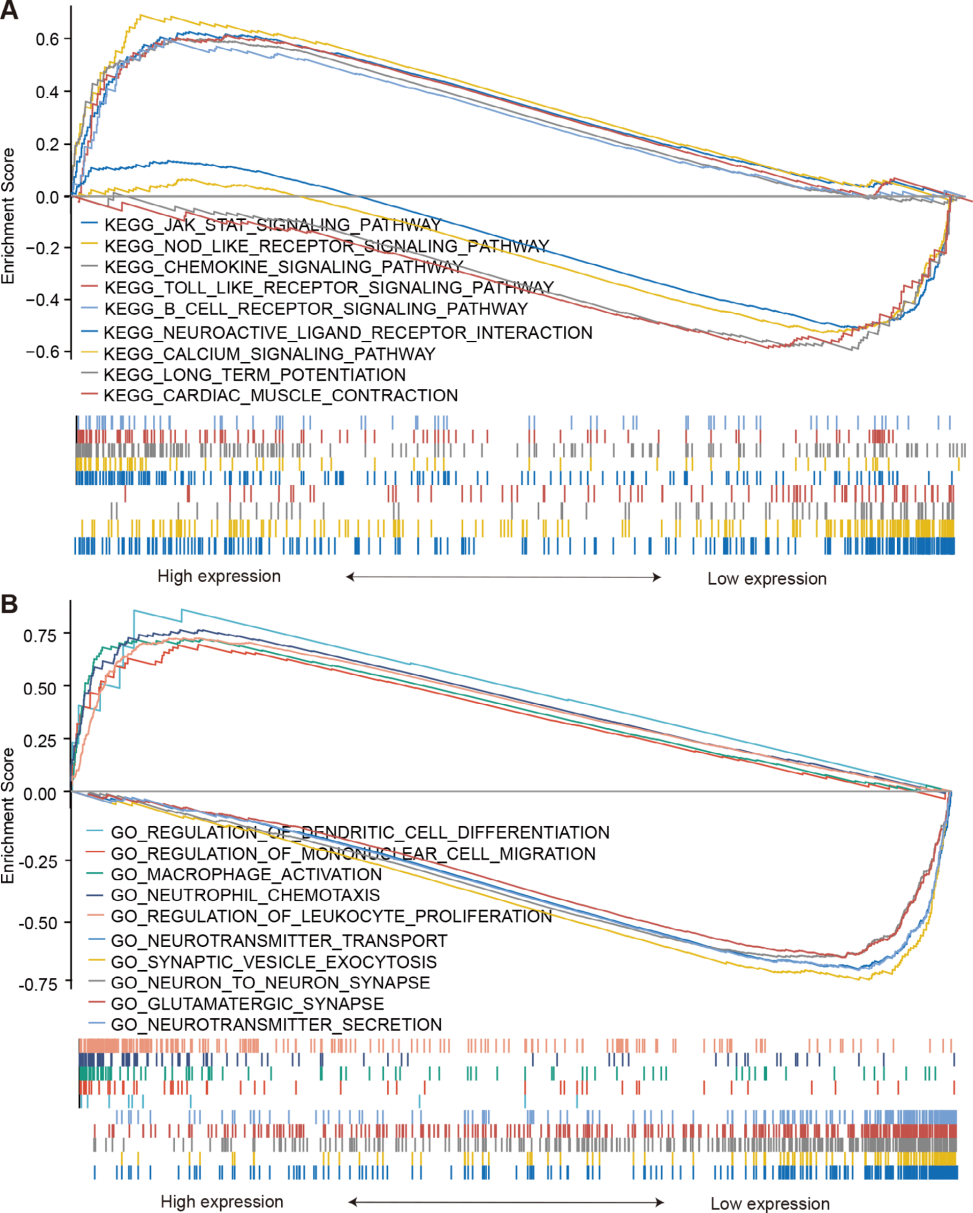
LILRB1 functional annotation in glioma. According to GSEA results, the top five KEGG pathways for the LILRB1^high^ include the toll-like receptor signaling pathway, B cell receptor signaling pathway, chemokine signaling pathway, JAK/STAT signaling pathway and NOD-like receptor signaling pathway. Calcium signaling pathway, cardiac muscle contraction, neuroactive ligand receptor interaction and long-term potentiation are the four KEGG pathways in LILRB1^low^ (**A**). GSEA findings showing varying levels of GO term enrichment in relation to LILRB1 expression. Top five GO keywords for LILRB1^high^-macrophage activation, regulation of dendritic cell differentiation, neutrophil chemotaxis, regulation of mononuclear cell migration and regulation of leukocyte proliferation. Top five GO keywords for LILRB1^low^- neurotransmitter transport, synaptic vesicle exocytosis, neuron to neuron synapse, glutamatergic synapse, neurotransmitter secretion (**B**). Based on FDR value, adjusted P value and NES, GSEA generated all of its results

Understanding the underlying functions of LILRB1 in the glioma formation and progression will be improved by the identification of associated genes. The TCGA dataset provided us with the LILRB1 associated genes. The heatmap of the top 50 genes of LRLRB1 that are connected favorably and negatively in glioma (Fig. 4A-B). Figure 4 C shows a volcano plot of the Pearson positive and negative associations of LRLRB1 in glioma. Using the Clusterprofiler package, bar graphs of GO terms and KEGG pathways of the coinciding differentially expressed genes were created in glioma (Fig. 4D). As illustrated in Fig. 4D, functional enrichment clustering of these genes demonstrated a high connection with neutrophil activation, leukocyte migration and T cell activation in BP; secretory granule membrane, the external side of the plasma membrane and presynapse in CC; receptor ligand activity, passive transmembrane transporter activity and channel activity in MF as well as Chemokine signaling pathway, NOD-like receptor signaling pathway and PI3K-Akt signaling pathway in KEGG. Moreover, the GSEA was used for distinguishing between LILRB1^high^ and LILRB1^low^ glioma in terms of GO and KEGG enrichment (P.adjust value < 0.05, FDR < 0.05). The top 5 prominent KEGG pathways in LILRB1^high^ glioma were the JAK/STAT signaling pathway, NOD-like receptor signaling pathway, chemokine signaling pathway, toll-like receptor signaling pathway and B cell receptor signaling pathway. In contrast, neuroactive ligand receptor interaction, the calcium signaling pathway, long-term potentiation and cardiac muscle contraction were considerably enriched in the LILRB1^low^ phenotype (Fig. 5A). Regulation of dendritic cell differentiation, regulation of mononuclear cell migration, macrophage activation, neutrophil chemotaxis and regulation of leukocyte proliferation were the abundantly expressed GO terms in the LILRB1^high^ phenotype, whereas neurotransmitter transport, synaptic vesicle exocytosis, neuron-to-neuron synapse, glutamatergic synapse and neurotransmitter secretion were abundantly expressed in the LILRB1^low^ phenotype (Fig. 5B). Table 4 provides a summary of the GO and KEGG components. LILRB1 is consequently connected to the emergence and spread of glioma.

**Table 4:** Gene enrichment analysis based on high and low LILRB1 expression phenotype

Gene set name	NES	p.adjust	FDR
High expression			
KEGG_JAK_STAT_SIGNALING_PATHWAY	2.127	0.002	0.002
KEGG_NOD_LIKE_RECEPTOR_SIGNALING_PATHWAY	2.083	0.002	0.002
KEGG_CHEMOKINE_SIGNALING_PATHWAY	2.068	0.002	0.002
KEGG_TOLL_LIKE_RECEPTOR_SIGNALING_PATHWAY	2.036	0.002	0.002
KEGG_B_CELL_RECEPTOR_SIGNALING_PATHWAY	1.860	0.002	0.002
GO_REGULATION_OF_DENDRITIC_CELL_DIFFERENTIATION	1.843	0.004	0.002
GO_REGULATION_OF_MONONUCLEAR_CELL_MIGRATION	2.038	0.004	0.002
GO_MACROPHAGE_ACTIVATION	2.327	0.004	0.002
GO_NEUTROPHIL_CHEMOTAXIS	2.472	0.004	0.002
GO_REGULATION_OF_LEUKOCYTE_PROLIFERATION	2.554	0.004	0.002
Low expression			
KEGG_NEUROACTIVE_LIGAND_RECEPTOR_INTERACTION	-2.165	0.008	0.005
KEGG_CALCIUM_SIGNALING_PATHWAY	-2.128	0.006	0.004
KEGG_LONG_TERM_POTENTIATION	-2.088	0.004	0.003
KEGG_CARDIAC_MUSCLE_CONTRACTION	-2.073	0.004	0.003
GO_NEUROTRANSMITTER_TRANSPORT	-2.858	0.009	0.006
GO_SYNAPTIC_VESICLE_EXOCYTOSIS	-2.785	0.007	0.005
GO_NEURON_TO_NEURON_SYNAPSE	-2.781	0.012	0.009
GO_GLUTAMATERGIC_SYNAPSE	-2.775	0.012	0.009
GO_NEUROTRANSMITTER_SECRETION	-2.770	0.007	0.005

NES: normalized enrichment score; P.adjust: adjusted P value; FDR: false discovery rate. Gene sets are deemed significant when their adjust P-value and FDR q-value < 0.05

### The protein-protein interaction network construction of LILRB1 and related genes in glioma

The PPI network of LILRB1 and related genes in glioma was created by Cytoscape (Additional file 1: Supplementary Figure S1A), and the top 10 hub genes of LILRB1 discovered by the CytoHubba plugin were ranked in terms of the NCC score (Additional file 1: Supplementary Figure S1B). There are positive correlations between LILRB1 and LILRB1-related hub genes including CD86, CD4, CD40, CD80, ITGAX, ITGAM, and CSF2 (Additional file 1: Supplementary Figure S1C-I).

### DNA methylation analysis of LILRB1 in glioma

Methylation is a significant epigenetic modification. We subsequently explored to determine if LILRB1 expression was connected to LILRB1 DNA methylation in LGG (Additional file 1: Supplementary Figure S2A) and GBM (Additional file 1: Supplementary Figure S2B). Methylation at sites detected by the following probes exhibited a negative connection with LILRB1 gene expression: cg02340056 (r = -0.339, P < 0.001), cg24154699 (r = -0.262, P < 0.001), cg13762704 (r = -0.243, P < 0.001), cg26778001 (r = -0.171, P < 0.001) in LGG and cg02340056 (r = -0.462, P < 0.001) in GBM, but a positive correlation with LILRB1 gene expression cg24154699(r = 0.379, P < 0.01), cg13762704(r = 0.296, P < 0.05) in GBM, no conclusive link with LILRB1 gene expression cg26778001(r = -0.142, P > 0.05) in GBM. Hypomethylation at cg02340056, cg24154699, cg13762704, and cg26778001 in the LILRB1 promoter was connected with worse prognosis. (Additional file 1: Supplementary Figure S3A-D).

### Relationship between LILRB1 expression and tumor mutational burden and microsatellite instability analysis in glioma

There was a strong connection between TMB and LILRB1, and an inverse relationship between MSI and LILRB1 in patients with glioma (Additional file 1: Supplementary Figure S3E-F).

### Relationship between LILRB1 expression and immune cells infiltration in glioma

**Fig. 6:**
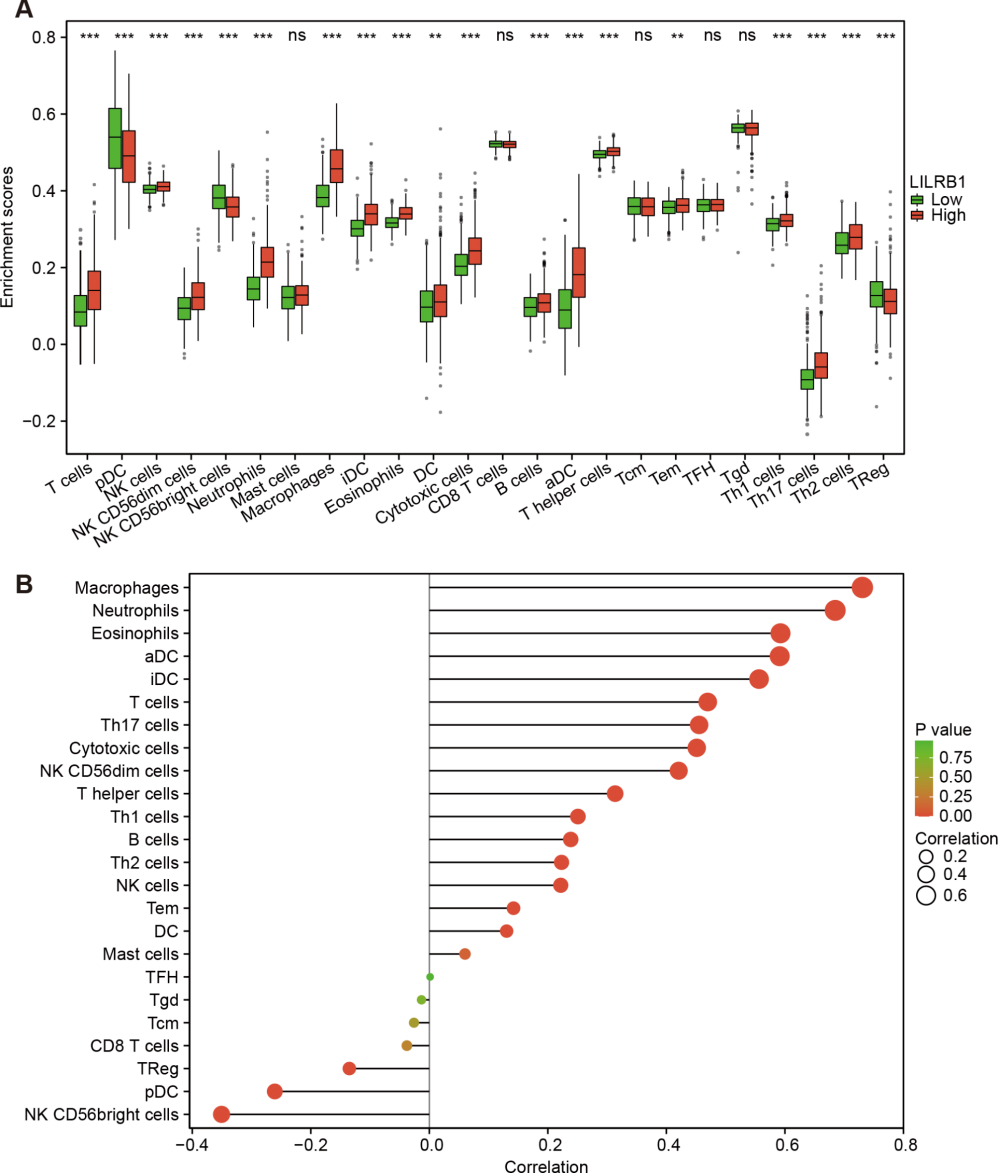
Immune infiltrates and LILRB1 expression in glioma are correlated. 24 tumor-infiltrating immune cell types and LILRB1 expression are correlated. (**A-B**). ns, P ≥ 0.05; *, P < 0.05; **, P < 0.01; ***, P < 0.001

**Fig. 7:**
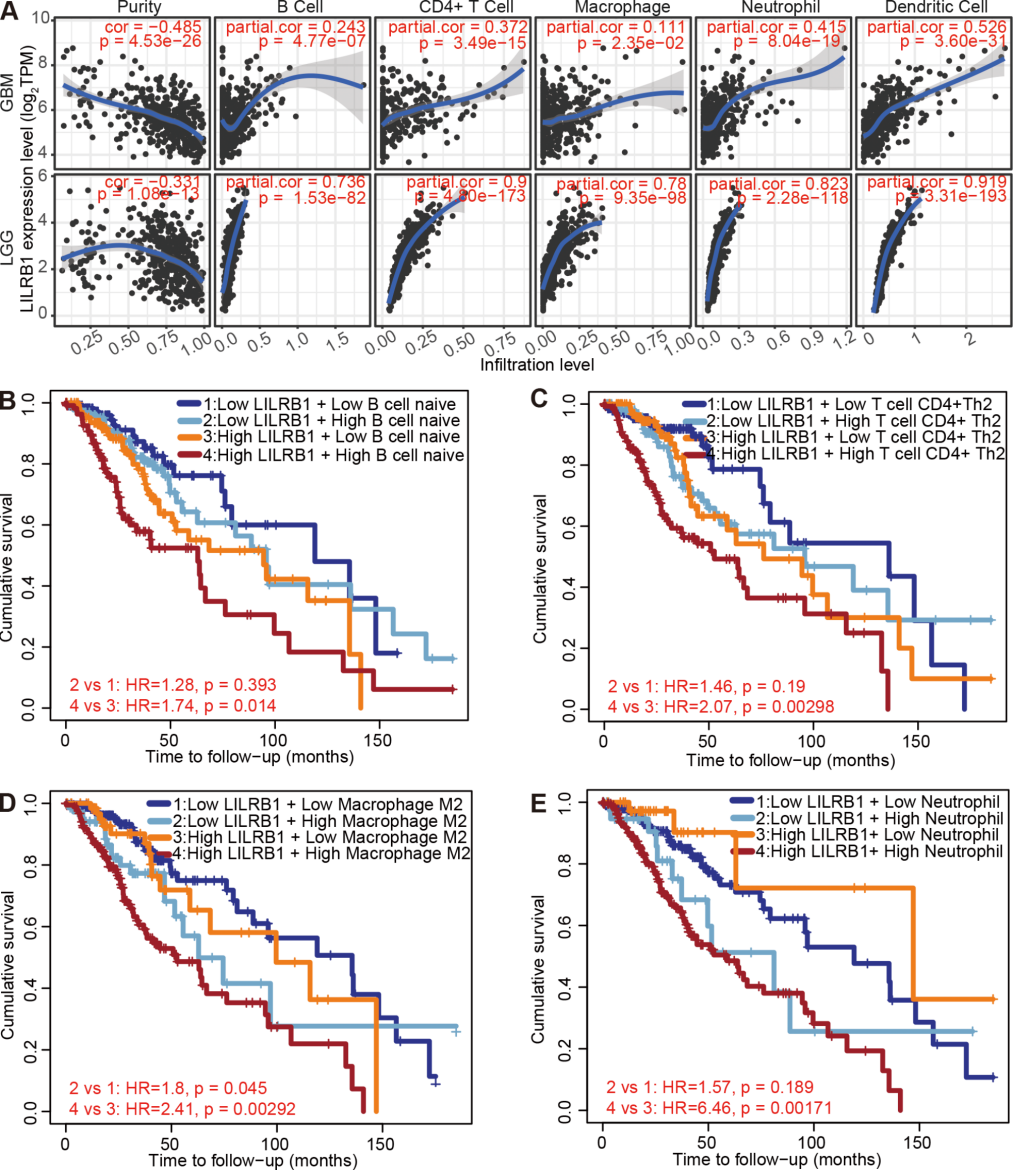
Immune infiltrates and LILRB1 expression in LGG and GBM are correlated. B cells, CD4^+^T cells, macrophages, neutrophils, and dendritic cells infiltrations were favorably linked with LILRB1 expression (**A**). A worse prognosis was associated with greater infiltration of B cells (**B**), CD4^+^T cells (**C**), M2 macrophages (**D**) and neutrophils (**E**)

The level of lymphocytic infiltration in tumor tissues in various neoplasms serves as a predictor of prognosis and condition of sentinel lymph nodes. Therefore, we investigated the relationship between immune cells that have invaded glioma and LILRB1 expression. LILRB1 expression linked strongly with T cells, plasmacytoid DCs(pDCs), NK cells, NK CD56bright cells, NK CD56dim cells, neutrophils, macrophages, immature DCs (iDCs), eosinophils, cytotoxic cells, B cells, activated DCs (aDCs), T helper cells, Th1 cells, Th17cells, Th2 cells, Treg (P < 0.001), DC and T effector memory (Tem) cells infiltration (P < 0.01) (Fig. 6A). Conversely, LILRB1 expression did not significantly correlate with mast cells, CD8 Tcells, T central memory (Tcm)cells, T follicular helper (Tfh) cells and Tgd infiltration. LILRB1 expression was favorably linked with macrophages, neutrophils, eosinophils, aDCs, iDCs, T cells, Th17cells, cytotoxic cells, NK CD56dim cells, T helper cells, Th1 cells, B cells, Th2 cells, NK cells, Tem cells and DC infiltration. Whereas, LILRB1 expression demonstrated significant adverse relationships with NK CD56bright cells, pDCs and Treg infiltration (Fig. 6B). Moreover, TIMER analysis showed a strong association between LILRB1 expression and infiltration of B cells (r = 0.736, P = 1.53e-82), CD4^+^T cells (r = 0.9, P = 2.80e-173), macrophages (r = 0.78, P = 9.35e-98), neutrophils (r = 0.823, P = 2.28e-118), and dendritic cells (r = 0.919, P = 3.31e-193) in LGG and B cells (r = 0.243, P = 4.77e-07), CD4^+^T cells (r = 0.372, P = 3.49e-15), macrophages (r = 0.111, P = 2.35e-02), neutrophils (r = 0.415, P = 8.04e-19) and dendritic cells (r = 0.526, P = 3.60e-31) in GBM (Fig. 7A). Additionally, TIMER2.0 demonstrated that a poor prognosis for LILRB1^high^ glioma was related with increased B cell infiltration (Fig. 7B; HR = 1.74, P = 0.014). Similarly, higher CD4^+^T cell, M2 macrophage, neutrophil (Fig. 7C-E) and myeloid dendritic cell (Additional file 1: Supplementary Figure S4) infiltration also correlated with worse outcome in glioma.

### Correlation between LILRB1 expression and M2 macrophage markers in glioma

Besides, we discovered that CCL22, CD163, CLEC7A, CLEC10A, CSF1R, FCGR3A, PTPRC, IRF1, PDCD1LG2, PDGFB, PPARG, IL-10, STAT6, TGFB1, IL23A and IRF4 of the M2 macrophage markers were found to have a positive correlation with LILRB1 expression in glioma (Additional file 1: Supplementary Figure S5A-P). Moreover, those of M2 macrophage markers were significantly overexpressed in glioma (Additional file 1: Supplementary Figure S6A-P). What is more, patients with glioma who expressed these M2 macrophage markers highly had visibly lower OS than those who did not (Additional file 1: Supplementary Figure S7A-P). The above results confirm the existence of a connection between LILRB1 and immune cells in the immune microenvironment. LILRB1 expression will affect the TME through M2 macrophages and may have a substantial effect on the immunological response of glioma.

### Correlation between the expression of LILRB1 and common immune checkpoints in glioma

We investigated the correlation between LILRB1 levels and those of typical ICPs in order to determine how LILRB1 expression impacts the response to immunotherapy. Examining the levels of BTLA, CD96, CD226, CD244, CD274, CTL4, HAVCR2, PDCD1LG2, and PDCD1 revealed that the high LILRB1 expression group had a high expression of ICPs (Additional file 1: Supplementary Figure S8A). In addition, we discovered a favorable connection between LILRB1 expression and overexpression in glioma of BTLA, CD96, CD226, CD244, CD274, CTL4, HAVCR2, PDCD1LG2, and PDCD1 (Additional file 1: Supplementary Fig. 8B-J). These higher ICP levels imply that individuals with high LILRB1 expression may have better immunotherapy.

### Univariate and multivariate Cox regression analysis of prognostic variables

**Fig. 8:**
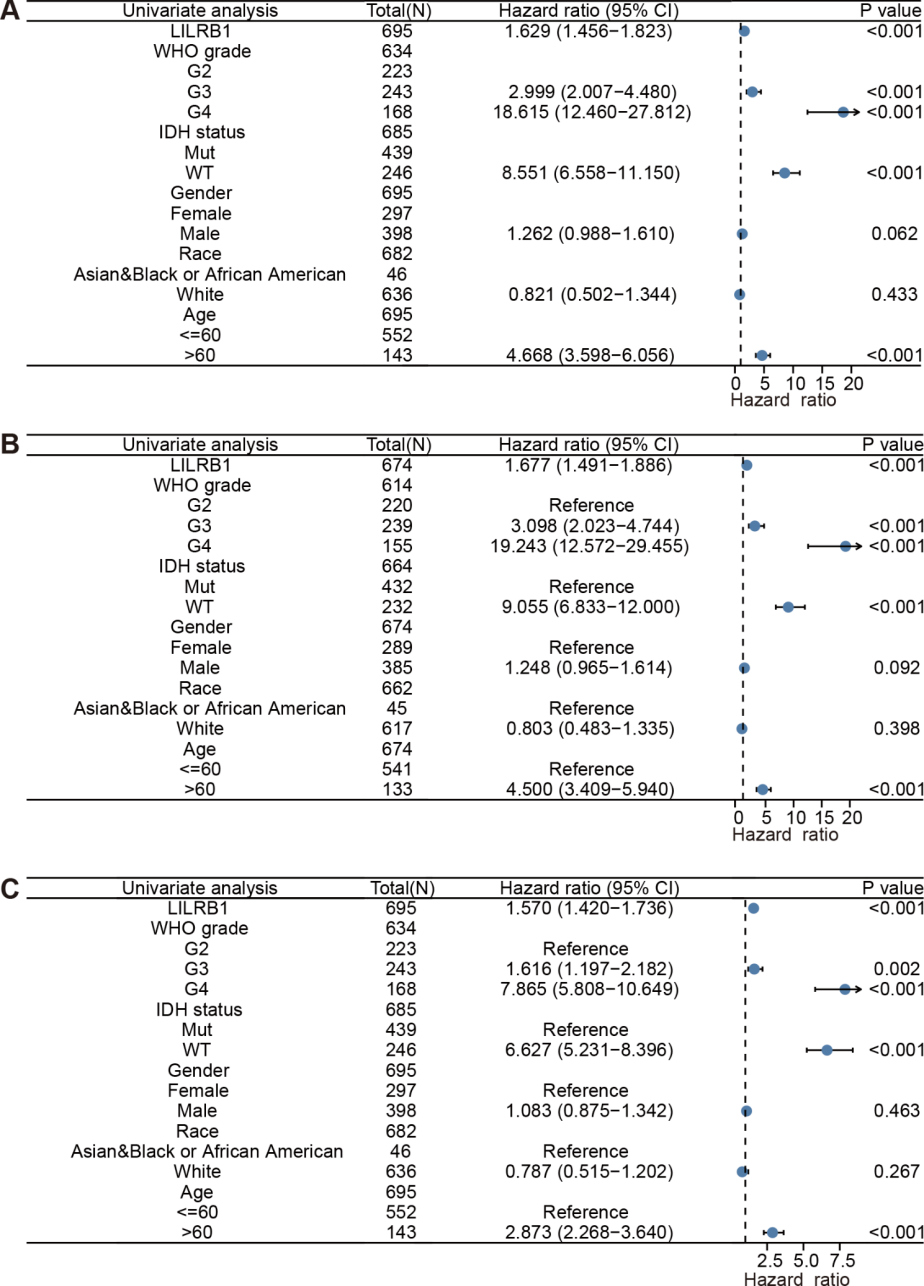
Univariate analysis of clinical pathological variables and LILRB1 expression. Univariate analysis of clinical pathological variables and LILRB1 expression for OS (**A**), DSS (**B**) and PFI (**C**). Covariates -LILRB1 expression, age, gender, race, WHO grade and IDH status

**Fig. 9:**
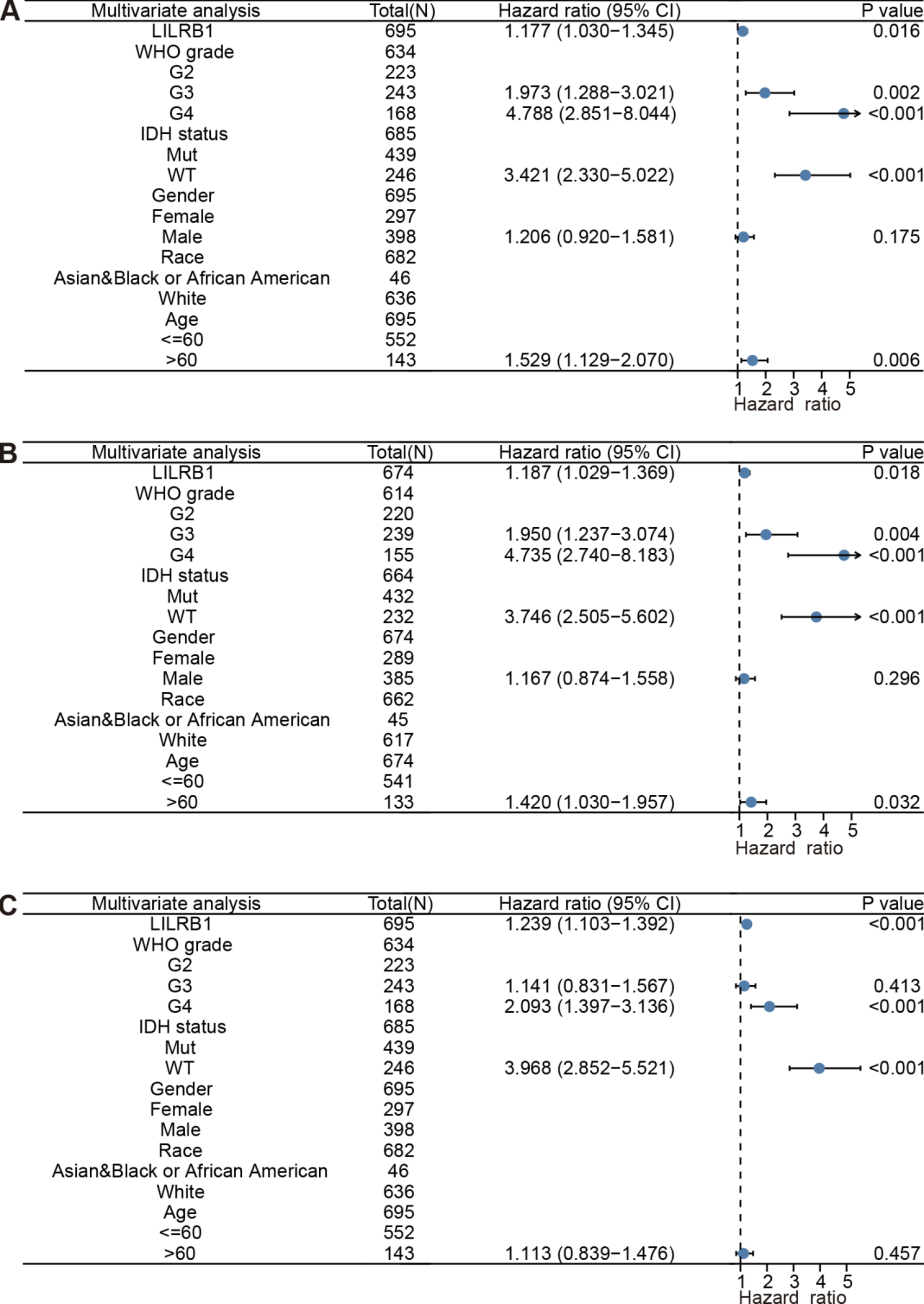
Multivariate Cox analysis of clinical pathological variables and LILRB1 expression. Multivariate Cox analysis of clinical pathological variables and LILRB1 expression for OS (**A**), DSS (**B**) and PFI (**C**). Covariates -LILRB1 expression, age, gender, race, WHO grade and IDH status

LILRB1 was demonstrated to be strongly connected with the OS (HR = 1.629, 95%CI = 1.456, 1.823, P < 0.001), DSS (HR = 1.677, 95%CI = 1.491, 1.886, P < 0.001) and PFI (HR = 1.570, 95%CI = 1.420, 1.736, P < 0.001) in univariate Cox regression analysis. Furthermore, the multivariate Cox regression analysis equally demonstrated that LILRB1 is a standalone risk factor for OS (HR = 1.177, 95% CI = 1. 030, 1.345, P = 0.016), DSS (HR = 1.187, 95%CI = 1.029, 1.369, P = 0.018) and PFI (HR = 1.239, 95%CI = 1.103, 1.392, P < 0.001). The results are summarized in Figs. 8 and 9.

### LILRB1 enhances proliferation, migration and invasion in glioma cells

**Fig. 10:**
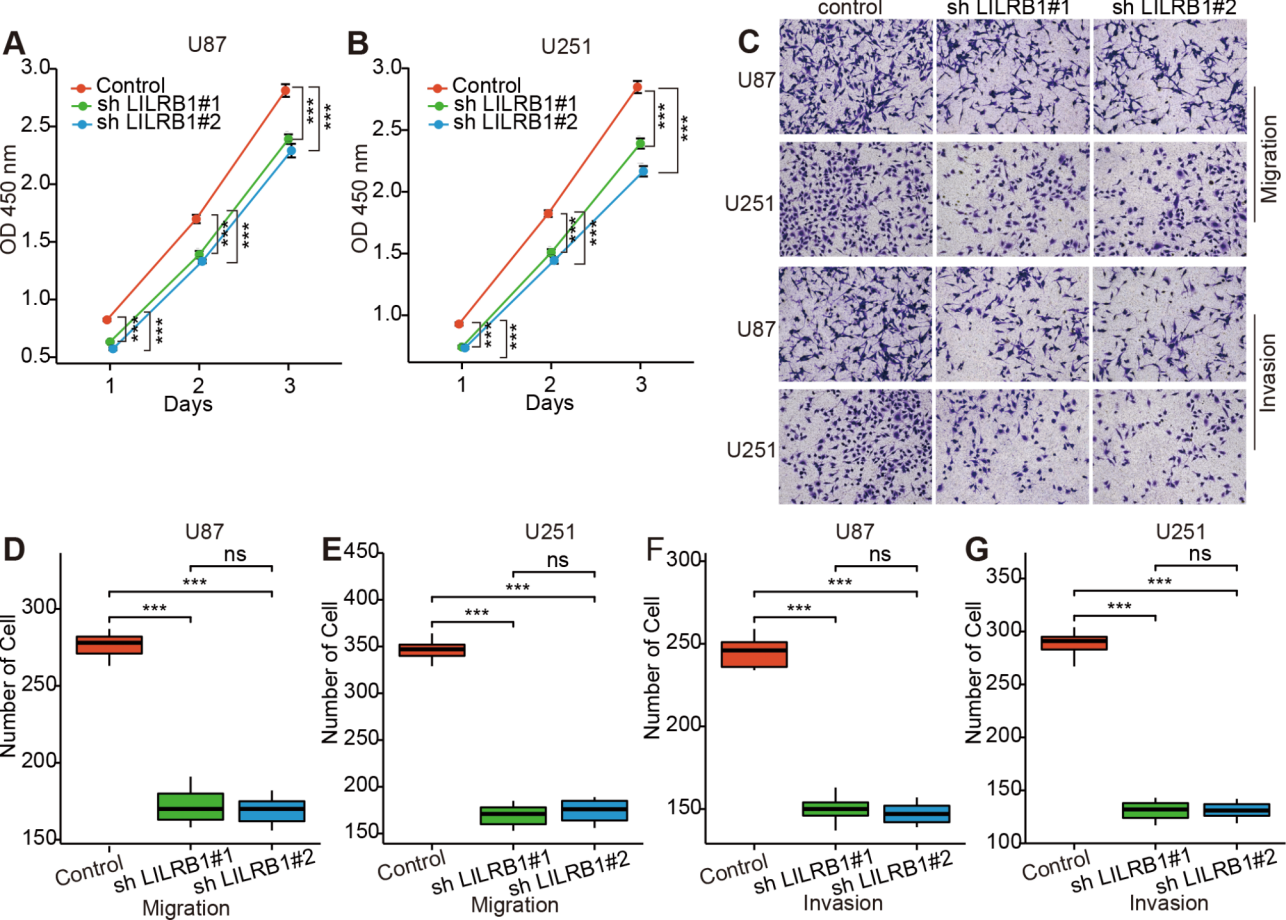
Vitro experiments. CCK-8 assay detected the proliferation rate of U87 (**A**) and U251 cells (**B**) after co-cultured with Control, sh LILRB1#1, sh LILRB1#2. Transwell results of the Control, sh LILRB1#1, sh LILRB1#2 effect on the migration and invasion of U87 and U251 cells (**C-G**). ns, P ≥ 0.05; ***, P < 0.001

In CCK-8 assay, the OD value of the sh LILRB1#1 and sh LILRB1#2 group decreased significantly 24 h, 48 and 72 h after cell co-culture for different groups. This indicates the proliferation-promoting effect of LILRB1 on U87 and U251 cells (Fig. 10A-B). After 48 h of cell co-culture inoculation in the transwell experiments, an average cell count of 5 randomly selected fields was collected under a 100× microscope. The cell count of the sh LILRB1#1 and sh LILRB1#2 groups decreased significantly compared with the control groups of U87 and U251 cells in migration and invasion (Fig. 10C-G).

### Connection between tumor spread distance, volumes and LILRB1 expression in MRI image

**Fig. 11:**
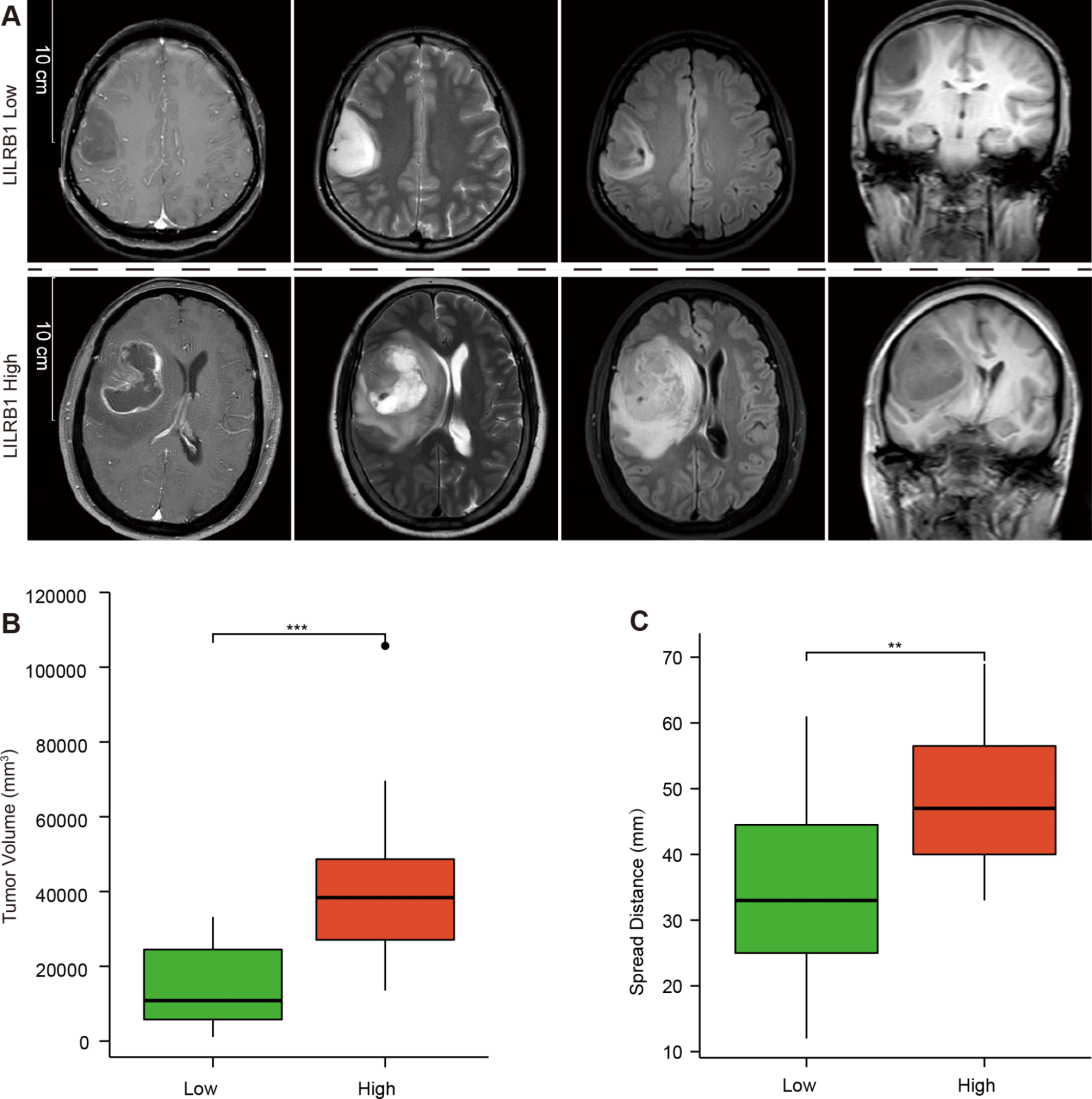
MRI images of typical glioma instances showing both low and high LILRB1 expression levels. Scale bars indicate 10 cm (**A**). Volumes of peritumoral T2WI abnormalities are distributed in low (n = 19) and high (n = 19) expression groups (**B**). LILRB1 levels are used to group the distribution of maximum spread distances (**C**). **, P < 0.01; ***, P < 0.001

Compared with low LILRB1 expression cases, glioma cases with high LILRB1 expression displayed greater volumes of peritumoral T2WI abnormality (Fig. 11A). Tumor volume and LILRB1 expression were shown to be significantly correlated (Fig. 11B). Moreover, there was a strong connection between tumor spread distance and LILRB1 expression (Fig. 11C).

## Discussion

The prominent receptor for HLA-G, LILRB1, is viewed as an immunosuppressive molecule. According to several researches, LILRB1 is essential for promoting tumor growth and metastasis, such as in breast [16], gastric [17] and pancreatic cancers [18]. Similarly, in our study, LILRB1 was found to be substantially connected with prognosis, WHO grade, IDH status and 1p/19q codeletion in glioma. In addition, univariate and multivariate Cox regression analyses detected LILRB1 as a standalone causal factor for glioma. Furthermore, in our experiments, LILRB1 was abundant in tumor tissues of patients with glioma and increased levels of LILRB1 expression were strongly linked to a worse prognosis. Last but not least, the MRI images confirmed that an elevated expression of LILRB1 was linked with a bigger tumor volume and a longer spread distance in patients with glioma. Therefore, LILRB1 may be a standalone factor in glioma with poor prognosis.

Glioma, lung, stomach, prostate, and colon cancers are only a few of the cancer types for which the JAK/STAT signaling pathway has been linked. Overactivation of this pathway has been coupled with multiple carcinogenesis, progression, invasion, and metastasis, according to previous research [19]. Tu Y et al. demonstrated a close positive association between JAK-1 and STAT-3 and overall survival of patients with glioma [20]. We functionally annotated LILRB1 in our work using GO terms and KEGG pathways, and discovered that increased LILRB1 expression was positively connected with the JAK/STAT signaling pathway, indicating that LILRB1 would promote the development of glioma through the JAK/STAT signaling pathway. NOD-like receptor family pyrin domain containing 3 (NLRP3) is a member of pattern recognition receptors. Inflammation-related carcinogenesis, angiogenesis, cancer cell stemness, and chemoresistance have all been shown to be significantly regulated by NOD-like receptor [21]. It is an essential part of the NLRP3 inflammasome and is connected to the development of numerous illnesses. In our research, we discovered a favorable correlation between high LILRB1 expression and the NOD-like receptor signal pathway. We suggest that a higher LILRB1 expression would enhance glioma development by targeting the NOD-like receptor signal pathway. In the same manner, we also found that to enhance development of glioma, a higher LILRB1 expression was implicated in the regulation of the B cell receptor signaling pathway, chemokine signaling pathway and Toll-like receptor signaling pathway.

Using STRING, we discovered and verified candidate proteins that interacted with the LILRB1. PPI network analysis revealed that LILRB1 interacts with the top 10 hub genes including IL-10, PTPRC, IL-6, CD86, CD4, CD40, CD80, ITGAX, ITGAM, and CSF2, which have been linked to tumor development in previous studies.

DNA methylation is one of the variables linked to tumor growth, with promoter methylation being the most studied at present [22]. Several studies have shown high levels of promoter methylation to be linked to reduced gene production or gene silence, and low levels of promoter methylation to be linked to increased gene expression [23, 24]. Naturally, consistent with previous reports, we discovered in our investigation that the hypomethylation of the LILRB1 probe was related with elevated LILRB1 expression; this was more obvious in LGG than in GBM. Furthermore, patients with LGG with hypermethylated promoter regions had a better prognosis. Therefore, future studies might concentrate on the consequences of specific LILRB1 methylation sites on gene expression and patient mortality, particularly in patients with LGG.

The efficacy of TMB, a biomarker for predicting immune responses, is useful in treating different cancers, including breast [25, 26], colorectal [27, 28], and lung cancers [29, 30]. Park et al. discovered that patients who had lower TMB reported poorer prognoses for survival compared to those with increased TMB in breast cancer [25]. Short repetitive segments in the genome are hypermutable due to faulty DNA mismatch repair, which is referred to as MSI [31]. MSI has been identified in multiple cancer types, including glioblastoma, endometrial, ovarian, gastric, and prostate cancers [32–34]. Undoubtedly, in our study, LILRB1 expression was found to be positively connected with TMB in glioma, while LILRB1 expression was adversely correlated with MSI. Combined with TMB and MSI, LILRB1 may be a promising predictor for the efficacy of immunotherapy in patients with glioma.

Previous studies have reported LILRB1 expression in multiple immune infiltration cells, including NK cells, macrophages, dendritic cells, B cells and T cells, eosinophils, and basophils [7, 35–39]. Young NT et al. discovered that LILRB1 modulated dendritic cell development and function, which had a negative impact on the proliferation of primary and memory T cells [3]. In order to inhibit tumor cells from being phagocytosed, Barkal AA et al. demonstrated that macrophages of overexpressed LILRB1 engage with MHC class I molecules on tumor cell surfaces [40]. Similar to this, we confirmed in our study that increased LILRB1 expression was linked to increased infiltration of B cells, CD4^+^T cells, M2 macrophages, neutrophils, and dendritic cells in patients with glioma. Immune cells that have invaded tumors have a significant effect on how cancers form and progress, and they can either work against or in favor of tumor growth [41]. Taken together, LILRB1 may have a detrimental effect on the prognosis of glioma.by regulating the immune microenvironment.

The TME, which consists of infiltrating immune cells, endothelial cells, stromal cells, cancer-associated fibroblasts and tumor cells, is crucial to the development of tumors [41]. Tumor-associated macrophages (TAMs) are the main cancer-related infiltration elements in the TME. Macrophages that infiltrate tumor tissue can polarize into either a pro-tumor M2 or an antitumor M1 fraction. Furthermore, M2 TAMs play a unique function in encouraging angiogenesis and tumor development as well as suppressing adaptive immunity. Typically, a higher density of M2 TAMs is strongly connected with a poorer clinical outcome in gastric [42], breast [43], lung cancers [44] and glioma [45]. In our work, we demonstrated a favorable relationship between M2 TAMs infiltration and increased LILRB1 expression in patients with glioma. We examined further at how LILRB1 affected the immunological microenvironment in patients with glioma. LILRB1 expression was favorably connected with cytokines that are associated with M2 macrophages, including CCL22, CSF1R, PDGFB, IL-10, TGFB1 and others. For instance, Curiel et al. showed that CCL22 generated by TAMs supported the development of an immunosuppressive microenvironment in human ovarian cancer [46]. Additionally, TAMs induced the secretion of the anti-inflammatory cytokines TGF-β and IL-10, which stimulated tumor growth. Furthermore, PDGF and TGF-β, proangiogenic growth factors derived from TAMs induced neovascularization [47]. Last but not least, Noy R et al. demonstrated that TAMs promote tumor cell invasion and metastasis through a paracrine loop that includes tumor induced growth factor CSF-1 and macrophage generated epidermal growth factor [48]. Most importantly, our investigation revealed that these molecules more abundantly expressed in tumor than normal tissues and were connected with worse survival in patients with glioma. Thus, we suggest that in combination with M2 TAMs, LILRB1 may promote tumor metastasis, invasion, angiogenesis, and tumor development in glioma.

One of the most important immunotherapies for the treatment of cancers is immune checkpoint blockade therapy, which changed the landscape of cancer treatment [49]. The immune checkpoint blockade therapy’s well-known targets include anti-CTLA and anti-PD-1/PD-L1 [50]. Overactivation of ICPs, which can prevent antigens from being delivered to T cells and can impair T cell immunological function and survival, is a prominent tactic employed by tumor cells to evade immune detection [51, 52]. Finding possible immune genes in gliomas as biomarkers may be beneficial to immune checkpoint blockade therapy. Consequently, we further examined the relationship between LILRB1 and ICPs expression level in our study and showed that LILRB1 was favorably connected with BTLA, CD96, CD226, CD244, CD274, CTL4, HAVCR2, PDCD1LG2, and PDCD1. These findings imply that the treatment of matched monoclonal antibodies targeting immunological regulatory sites may be beneficial for patients with glioma that have elevated LILRB1 expression to avoid T cell depletion and reestablish anti-tumor immune response. These findings indicate that LILRB1 is a promising immune system gene in glioma that can influence the response to immunotherapy.

In our vitro experiments, the CCK-8 assay demonstrated that LILRB1 positively turbocharged the proliferation in glioma cells. Moreover, transwell assays determined that LILRB1 positively enhanced the migration and invasion in glioma cells. Finally, the MRI images demonstrated a positive association between high LILRB1 expression and a bigger tumor volume and a longer spread distance in patients with glioma. However, this study has some limitations that ought to be considered. More in vitro and in vivo functional investigations are required to confirm the mechanistic significance of LILRB1 in the genesis and development of glioma. Nevertheless, we establish that LILRB1 corresponds with immune infiltration in glioma and may be an oncogene and connects with immune infiltration in glioma and may be a therapeutic strategy and prognostic indicator for glioma.

## Conclusions

In the present study, we discovered that LILRB1 was markedly increased in glioma and was identified as a standalone risk factor for glioma. We establish that LILRB1 may be an oncogene and correlates with immune infiltration in glioma and may serve as a therapeutic target and prognostic indicator for glioma.

## Electronic supplementary material

Below is the link to the electronic supplementary material.

Supplementary Material 1

Supplementary Material 2

## Data Availability

The databases from which the study’s data were sourced were all openly accessible. Below is a list of the databases that were used during the inquiry. The UCSC XENA (https://xenabrowser.net/datapages/) ( TCGA and GTEx ), The Cancer Genome Atlas (TCGA) (https://portal.gdc.cancer.gov/) (TCGA-GBM and TCGA-LGG) database, Chinese Glioma Genome Atlas (CGGA) (http://www.cgga.org.cn/analyse/RNA-data.jsp), KEGG pathways (https://www.kegg.jp/kegg/kegg1.html), Molecular Signatures database (MSigDB) (https://www.gsea-msigdb.org/gsea/msigdb/index.jsp) (C2.CP), STRING database (https://string-db.org), MEXPRESS (https://mexpress.be), MethSurv program (https://biit.cs.ut.ee/methsurv), TIMER database (https://cistrome.shinyapps.io/timer/) and TIMER2.0 (http://timer.cistrome.org). The original contributions made for the study are contained in the article/supplementary material; any additional inquiries should be sent to the corresponding author.

## Abbreviations

LILRB1: Leukocyte immunoglobulin-like receptor subfamily B 1
GBM: Glioblastoma
LGG: Low-grade gliomas
WHO: World Health Organization
OS: Overall survival
CGGA: The Chinese Glioma Genome Atlas
NES: Normalized enrichment score
MF: Molecular function
HLA-G: Human leukocyte antigen G
HR: Hazard ratios
CI: Confidence intervals
GO: Gene Ontology
TCGA: The Cancer Genome Atlas
BP: Biological process
GSEA: Gene set enrichment analysis
TMB: Tumor Mutational Burden
CC: Cellular component
FBS: Fetal bovine serum
KEGG: Kyoto Encyclopedia of Genes and Genomes
TME: Tumor microenvironment
FDR: False discovery rate
ssGSEA: Single-sample Gene Set Enrichment Analysis
NCC: Normalized cross correlation
ICPs: Immune checkpoints
IDH: Isocitrate dehydrogenase
iDCs: Immature DCs
DSS: Disease-specific survival
DMEM: Dulbecco’s Modified Eagle Medium
DCs: Dendritic cells
Tem: T effector memory
PFI: Progression-free interval
pDCs: Plasmacytoid
TAMs: Tumor associated macrophages
MSI: Microsatellite instability
Tcm: T central memory
aDCs: Activated DCs
Tfh: T follicular helper
