# Supplementary material for: Elevated LILRB1 expression predicts poor prognosis and is associated with tumor immune infiltration in patients with glioma
Source: BMC Cancer. 2023 May 4;23:403. doi: 10.1186/s12885-023-10906-2 (PMC10161664; doi:10.1186/s12885-023-10906-2)
Supplement: Supplementary file 1 — Supplementary Material 1 [file 12885_2023_10906_MOESM1_ESM.pdf]

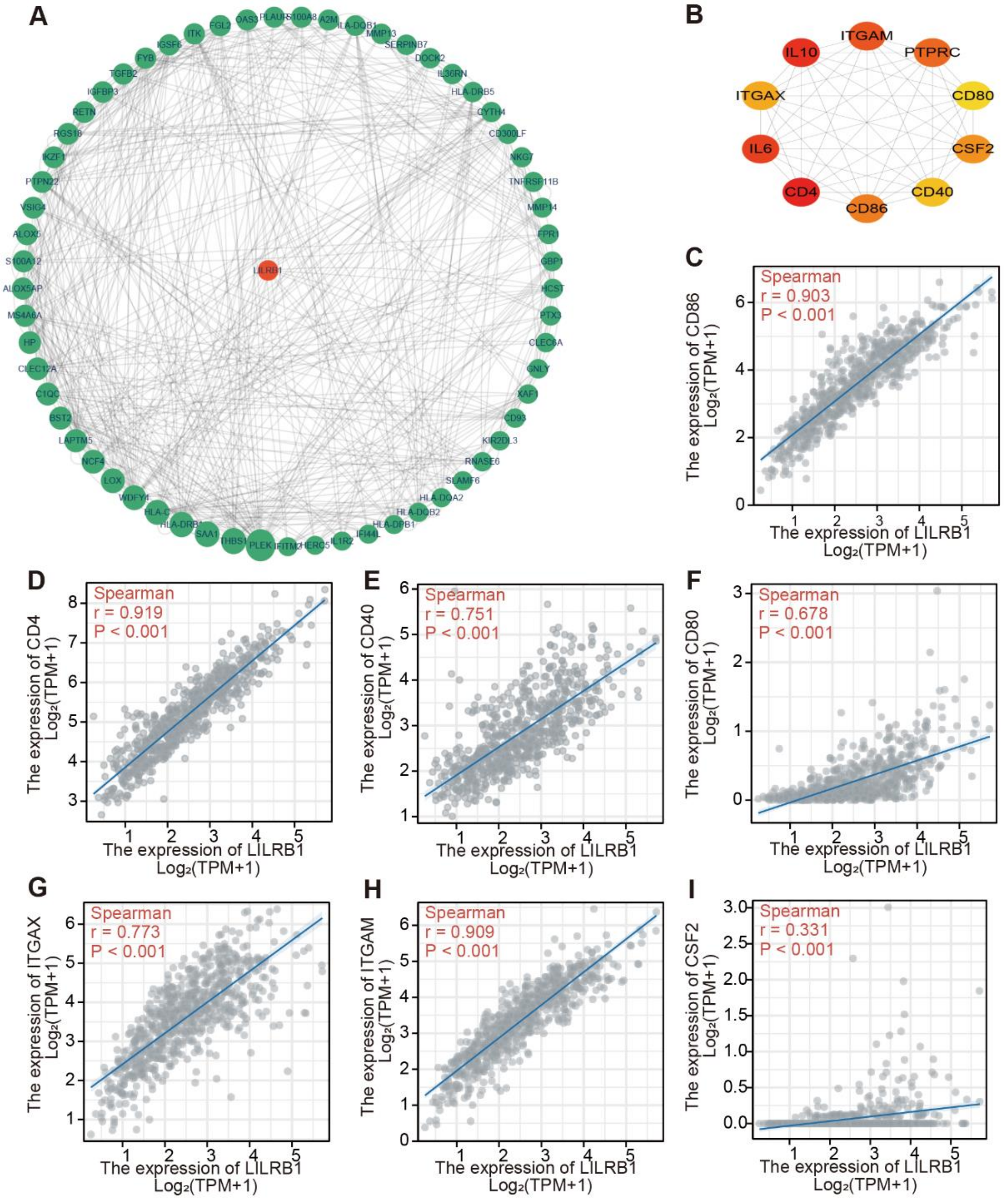

Supplementary Figure S1. PPI network and hub genes identification. LILRB1 and associated genes form a PPI network in glioma(**A**). 10 top hub genes of LILRB1 (**B**). Correlation between LILRB1 and LILRB1-related hub genes including CD86(**C**), CD4(**D**), CD40(**E**), CD80(**F**), ITGAX(**G**), ITGAM(**H**), CSF2(**I**).

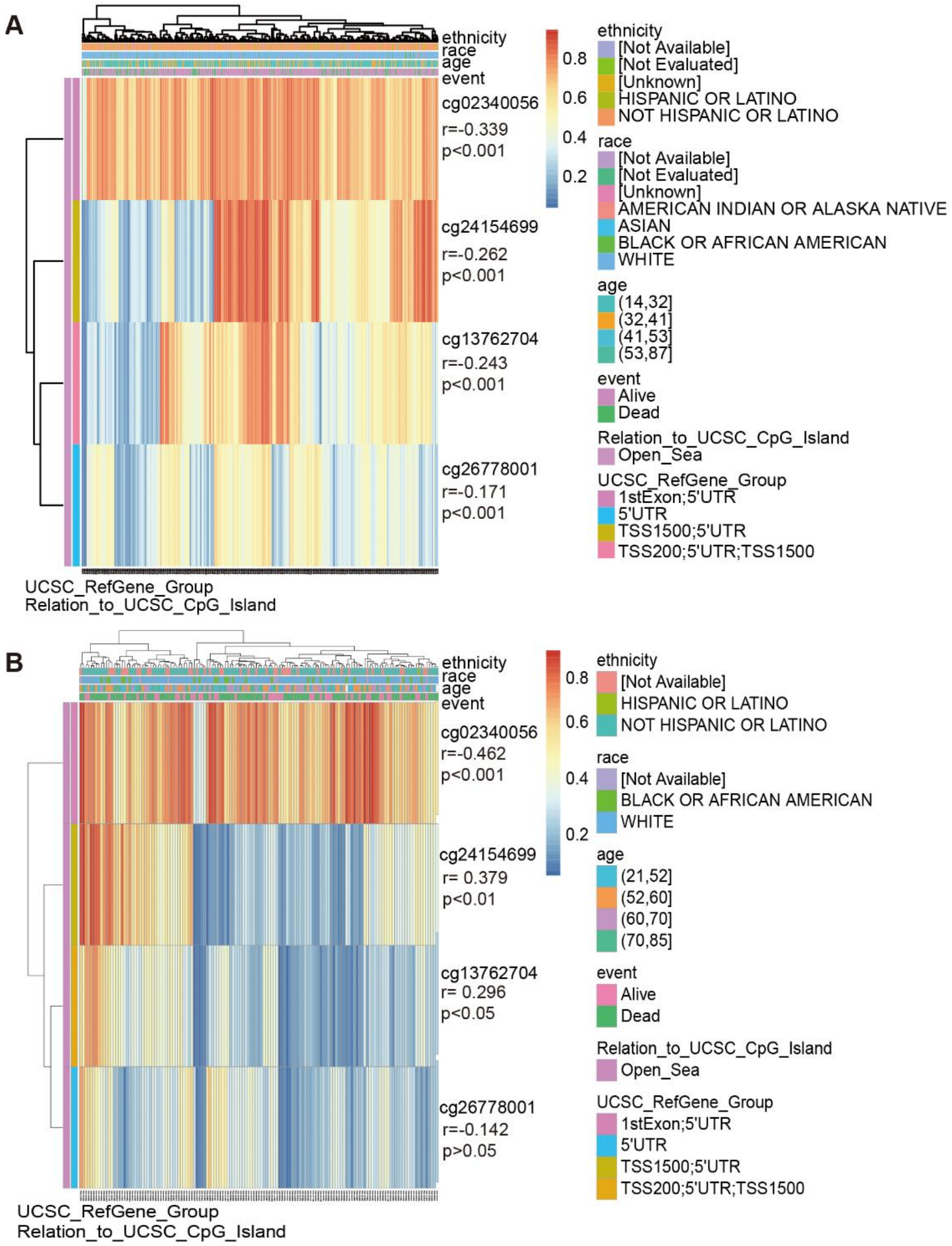

Supplementary Figure S2. Analysis of LILRB1 methylation in glioma. Analysis of LILRB1 methylation in LGG (**A**) and GBM(**B**). The methylation status of the LILRB1 gene is shown in a waterfall plot. Analysis is done on the relationships between LILRB1 expression and methylation.

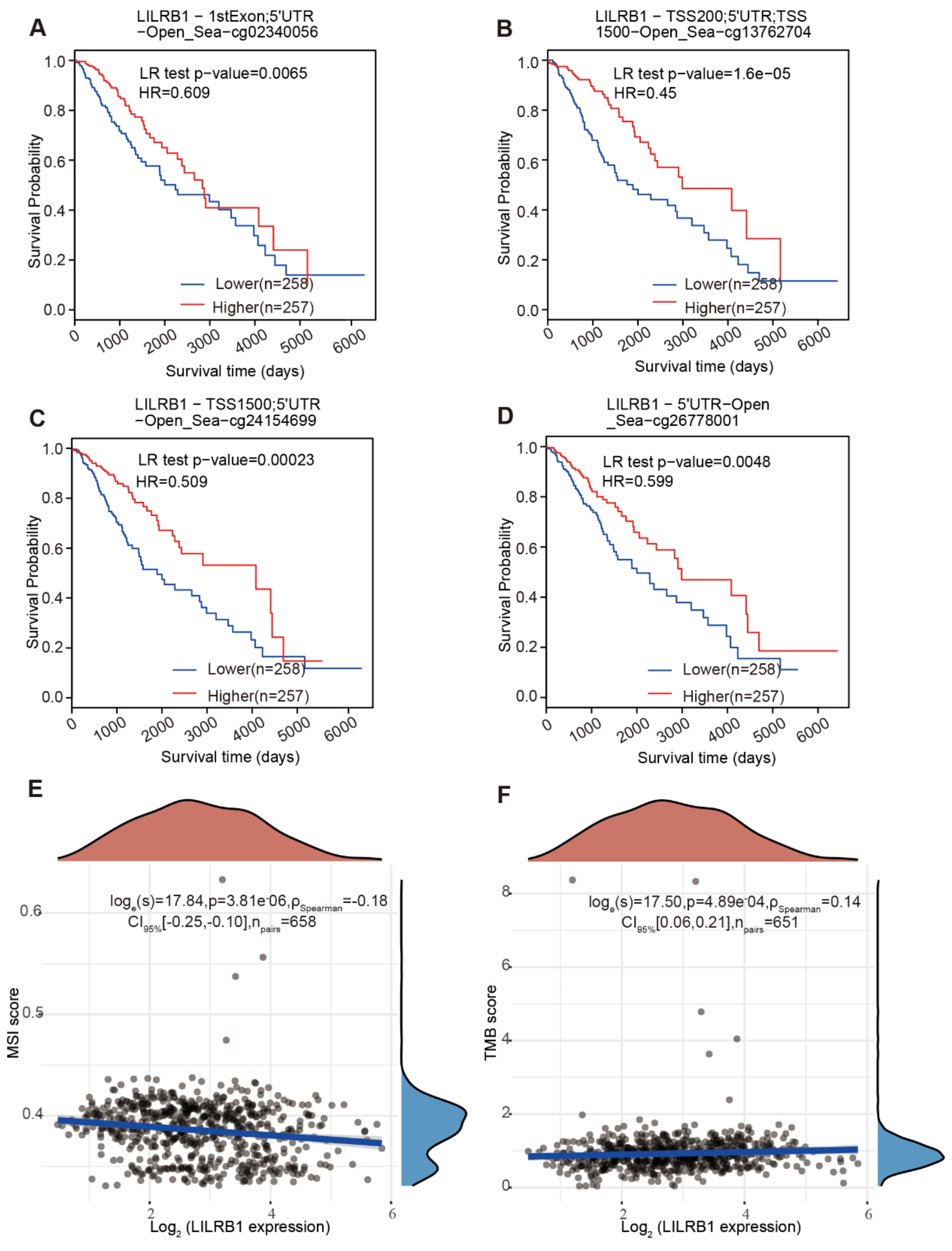

Supplementary Figure S3. Survival Analysis of LILRB1 expression and methylation. LILRB1 survival study based on several sites of methylation (**A-D**); $P < 0.05$ was regarded as statistically significant. Correlation between LILRB1 expression and MSI score (**E**), TMB score (**F**) in glioma.

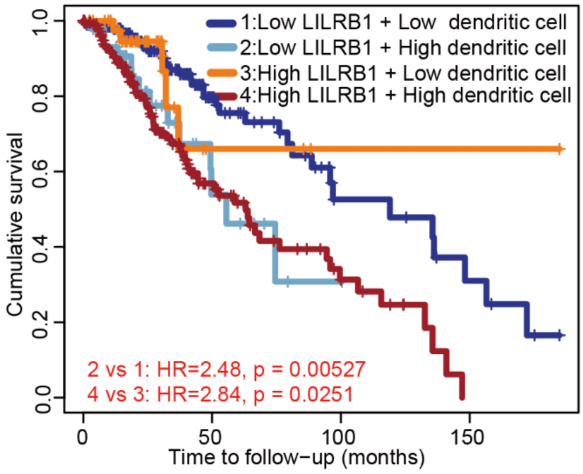

Supplementary Figure S4. Cumulative survival analysis of LILRB1 expression and myeloid dendritic cells infiltration in glioma. A worse prognosis was associated with greater infiltration of myeloid dendritic cells in glioma.

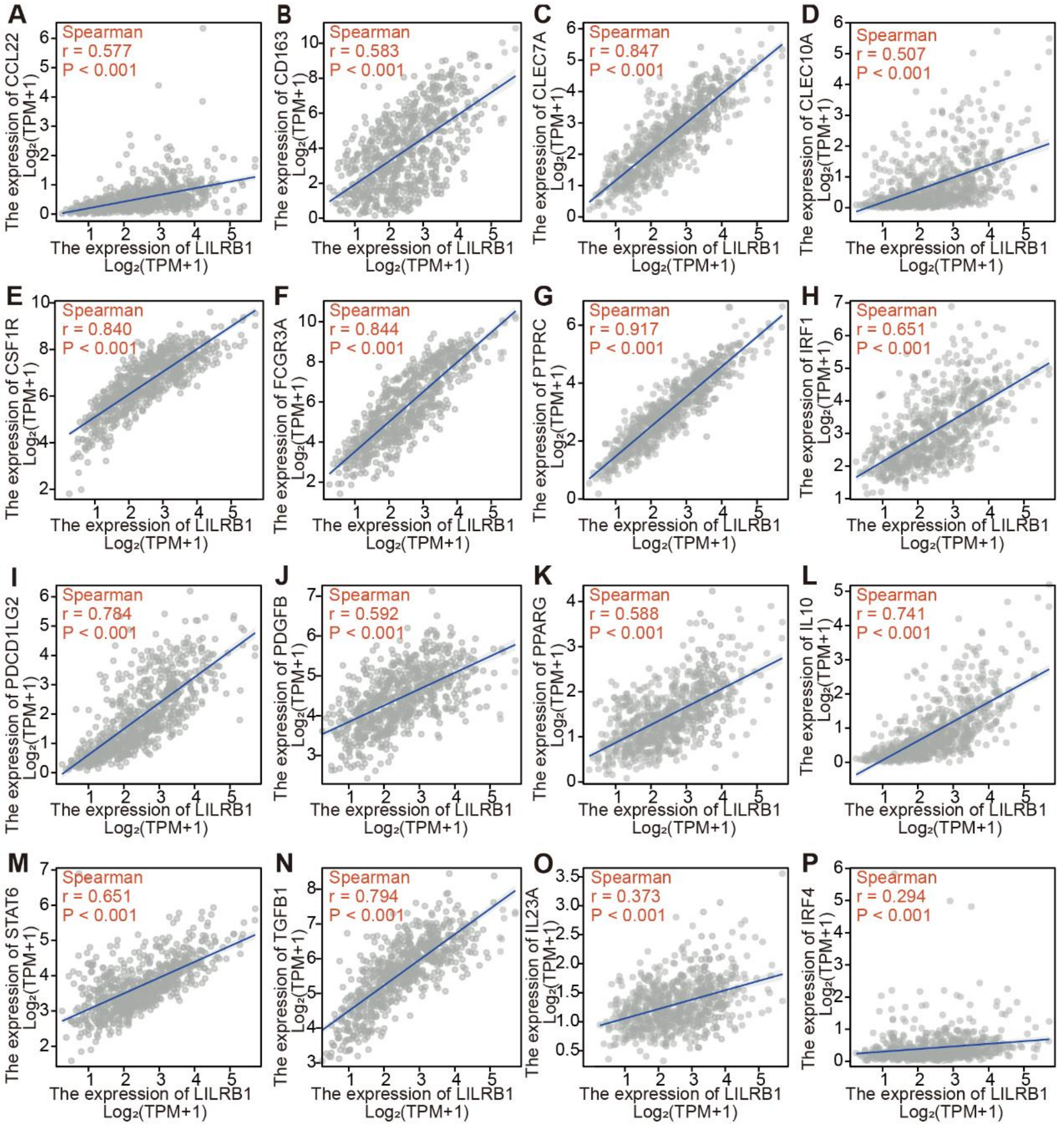

Supplementary Figure S5. LILRB1 expression and M2 macrophages markers in glioma are correlated. LILRB1 expression was positively correlated with CCL22 (**A**), CD163(**B**), CLEC7A (**C**), CLEC10A (**D**), CSF1R (**E**), FCGR3A (**F**), PTPRC(**G**), IRF1(**H**), PDCD1LG2(**I**), PDGFB(**J**), PPARG(**K**), IL-10(**L**), STAT6(**M**), TGFB1(**N**), IL23A(**O**), and IRF4(**P**).

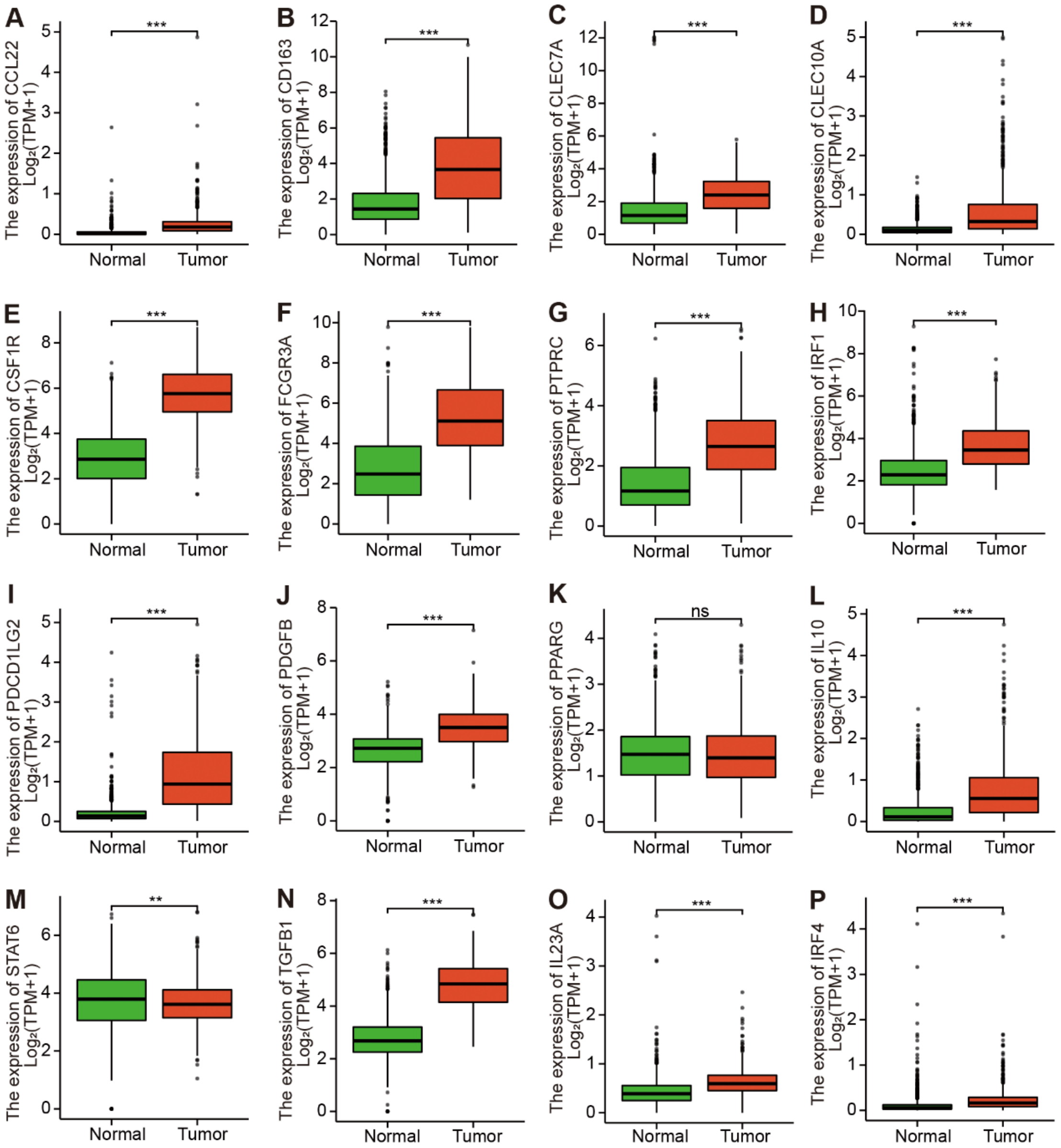

Supplementary Figure S6. The mRNA expression of M2 macrophages markers between cancer tissues and normal tissues. The mRNA expression of CCL22 (**A**), CD163(**B**), CLEC7A (**C**), CLEC10A (**D**), CSF1R (**E**), FCGR3A (**F**), PTPRC (**G**), IRF1(**H**), PDCD1LG2(**I**), PDGFB(**J**), PPARG(**K**), IL-10(**L**), STAT6(**M**), TGFB1(**N**), IL23A(**O**) and IRF4(**P**) between cancer tissues and normal tissues in patients with glioma in TCGA. ns, $P \geq 0.05$; *, $P < 0.05$; **, $P < 0.01$; ***, $P < 0.001$

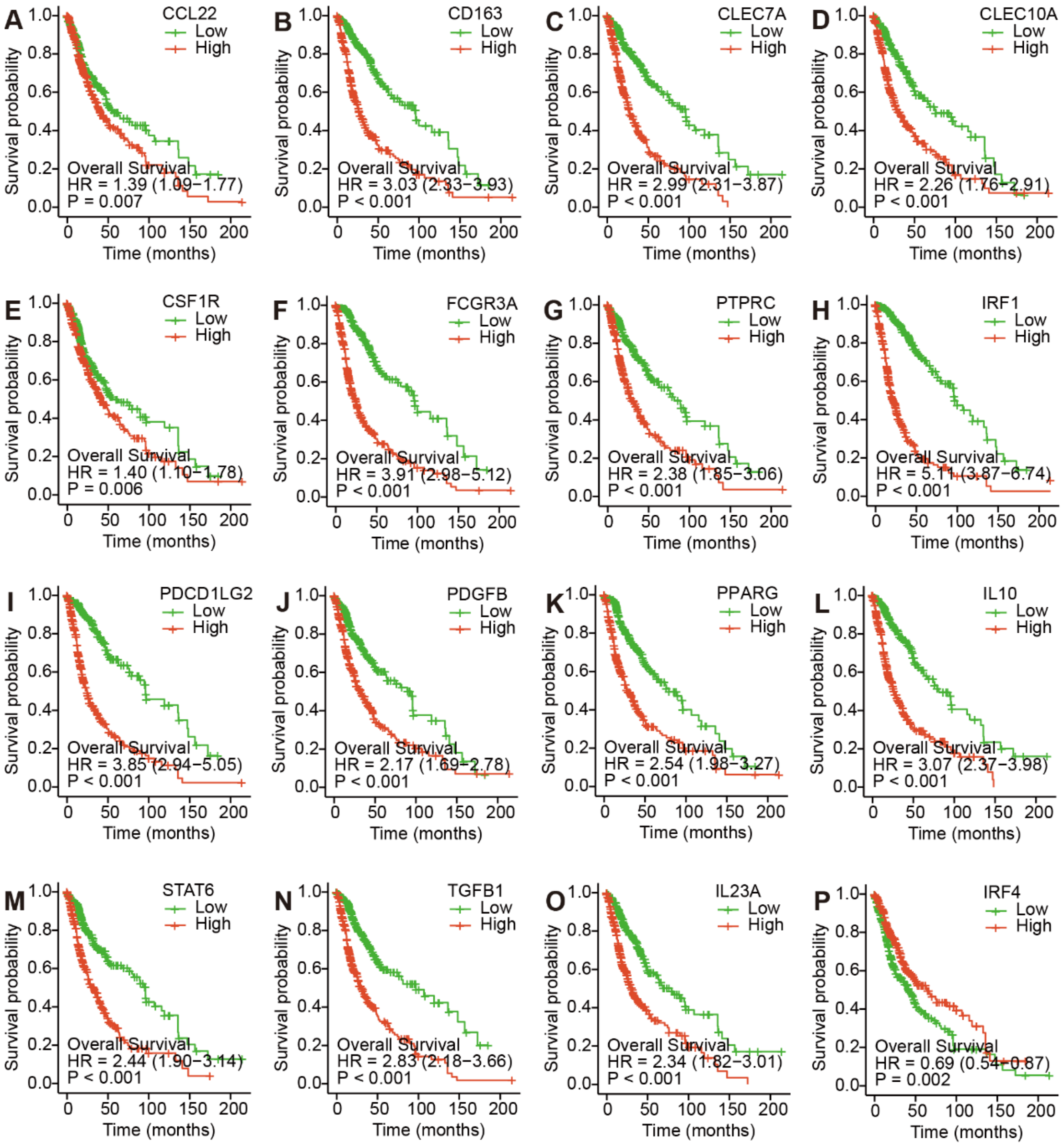

Supplementary Figure S7. Survival Analysis of LILRB1 expression and M2 macrophages markers. Comparison of the OS time between patients with glioma with high and low expression of CCL22 (**A**), CD163 (**B**), CLEC7A (**C**), CLEC10A (**D**), CSF1R (**E**), FCGR3A (**F**), PTPRC (**G**), IRF1 (**H**), PDCD1LG2 (**I**), PDGFB (**J**), PPARG (**K**), IL-10 (**L**), STAT6 (**M**), TGFB1 (**N**), IL23A (**O**) and IRF4 (**P**).

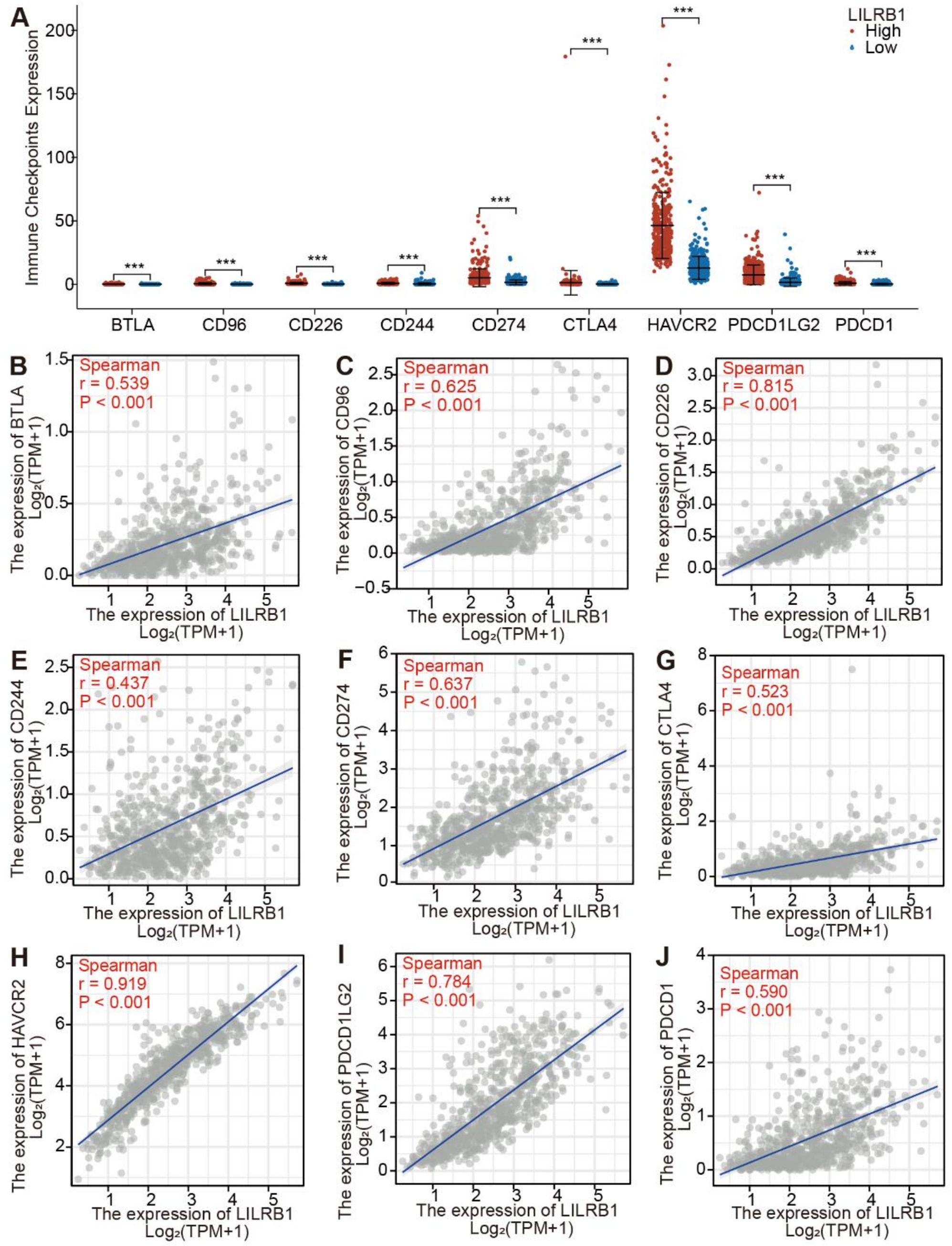

Supplementary Figure S8. Immune checkpoints. Connection between common immune checkpoints (ICPs) and the expression levels of LILRB1 (**A**). Spearman correlation coefficients for the association between the expression levels of LILRB1 and above ICPs(**B-J**). ***, P < 0.001.
