# Supplementary material for: Elevated LILRB1 expression predicts poor prognosis and is associated with tumor immune infiltration in patients with glioma
Source: BMC Cancer. 2023 May 4;23:403. doi: 10.1186/s12885-023-10906-2 (PMC10161664; doi:10.1186/s12885-023-10906-2)

LILRB1 for Fig.3D

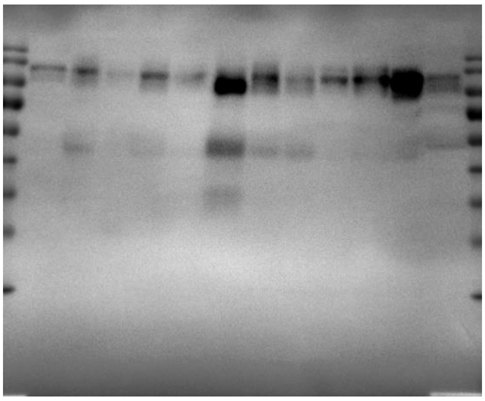

GAPDH for Fig.3D

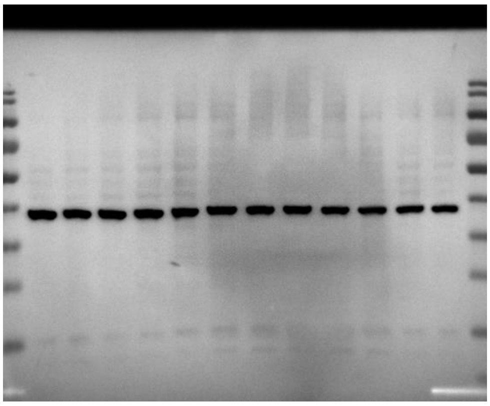

LILRB1 for Fig.3D

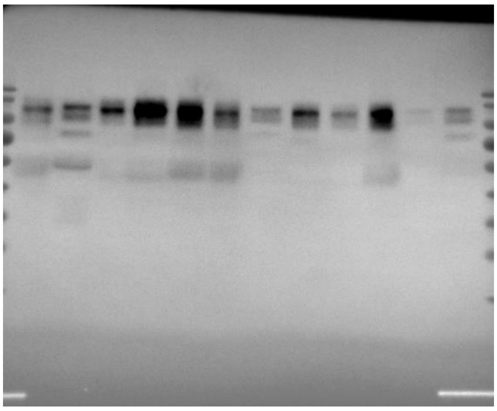

GAPDH for Fig.3D

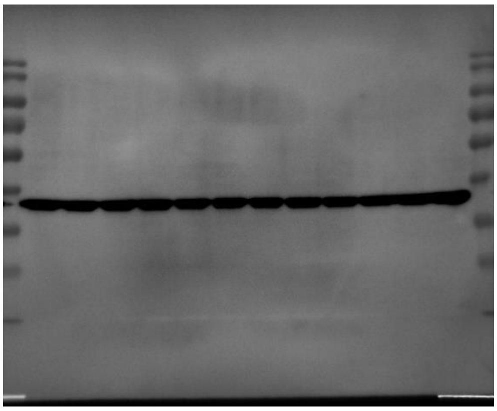

LILRB1 for Fig.3D

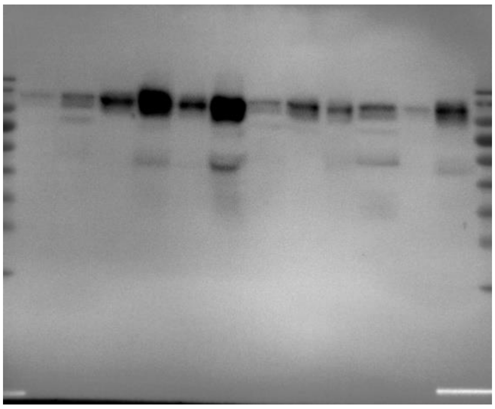

GAPDH for Fig.3D

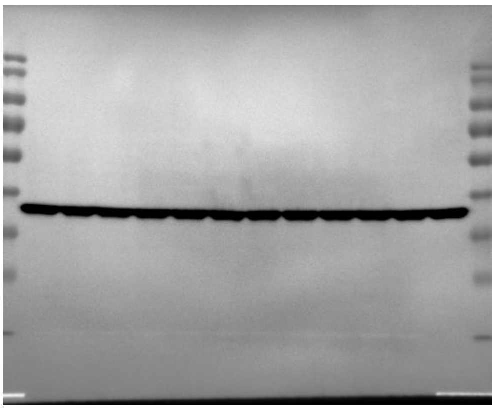

LILRB1 for Fig.3D

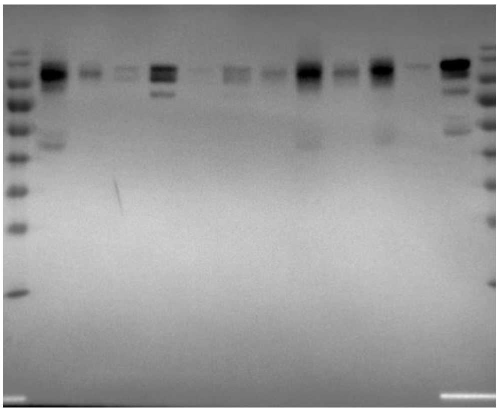

GAPDH for Fig.3D

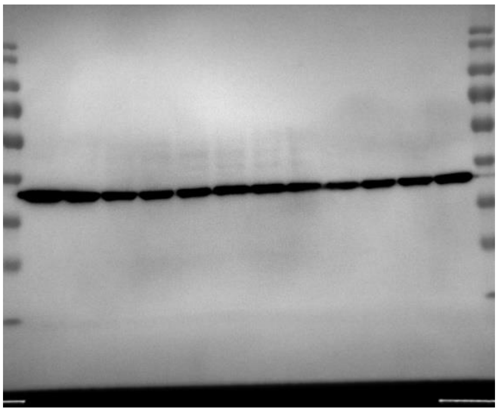

Supplement: Supplementary file 2 — Supplementary Material 2 [file 12885_2023_10906_MOESM2_ESM.pdf]
